# Advancing Enzyme’s Stability and Catalytic Efficiency through Synergy of Force-Field Calculations, Evolutionary Analysis, and Machine Learning

**DOI:** 10.1021/acscatal.3c02575

**Published:** 2023-09-11

**Authors:** Antonin Kunka, Sérgio M. Marques, Martin Havlasek, Michal Vasina, Nikola Velatova, Lucia Cengelova, David Kovar, Jiri Damborsky, Martin Marek, David Bednar, Zbynek Prokop

**Affiliations:** † Loschmidt Laboratories, Department of Experimental Biology and RECETOX, Faculty of Science, Masaryk University, Brno 601 77, Czech Republic; ‡ International Clinical Research Center, St. Anne’s University Hospital, Brno 601 77, Czech Republic

**Keywords:** biocatalysis, computational design, FireProt, machine learning, PROSS, protein engineering, stabilization, thermostability

## Abstract

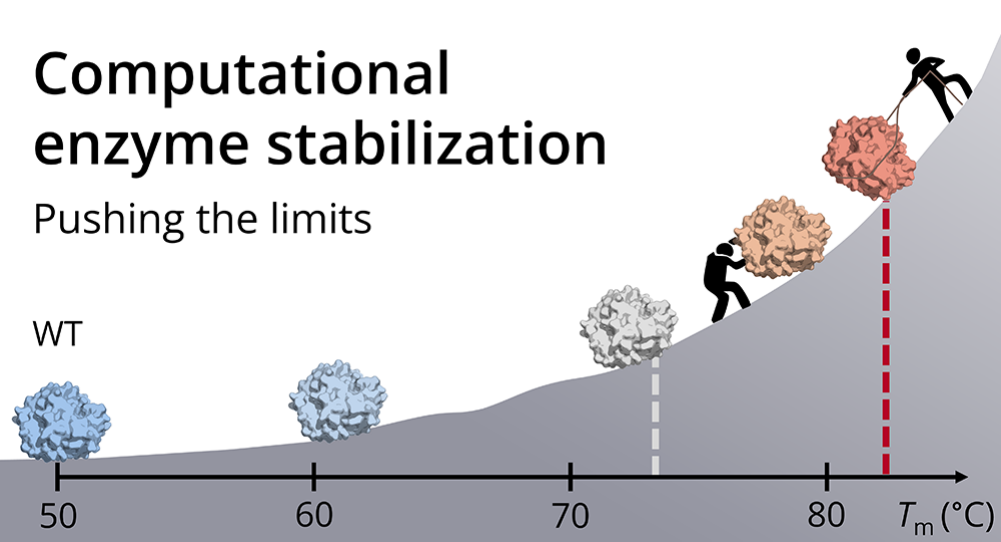

Thermostability is an essential requirement for the use of enzymes in the bioindustry. Here, we compare different protein stabilization strategies using a challenging target, a stable haloalkane dehalogenase DhaA115. We observe better performance of automated stabilization platforms FireProt and PROSS in designing multiple-point mutations over the introduction of disulfide bonds and strengthening the intra- and the inter-domain contacts by *in silico* saturation mutagenesis. We reveal that the performance of automated stabilization platforms was still compromised due to the introduction of some destabilizing mutations. Notably, we show that their prediction accuracy can be improved by applying manual curation or machine learning for the removal of potentially destabilizing mutations, yielding highly stable haloalkane dehalogenases with enhanced catalytic properties. A comparison of crystallographic structures revealed that current stabilization rounds were not accompanied by large backbone re-arrangements previously observed during the engineering stability of DhaA115. Stabilization was achieved by improving local contacts including protein–water interactions. Our study provides guidance for further improvement of automated structure-based computational tools for protein stabilization.

## Introduction

Computational design is a rapidly growing field that utilizes different techniques to stabilize enzymes for a wide range of applications.^1−4^ Biocatalysis represents a prime example of industrial sectors with great demand for protein thermostability. Operating at higher temperatures is highly advantageous since (i) the rate of chemical reaction approximately doubles every 10 °C, (ii) substrate diffusion is higher due to the lower viscosity of the medium, (iii) the solubility of molecules increases, and (iv) there is a lower risk for potential contamination.^5^ Improving protein stability has, therefore, been one of the major tasks in protein engineering for decades.

The stabilization strategies cover a wide range of methods based on the calculation of protein free energy,^6^ utilization of the statistical,^7^ empirical,^8^ or physical energy functions,^9^ bioinformatic approaches based on the consensus design^10^ or ancestral sequence reconstruction,^11^ and contemporary machine learning (ML) algorithms.^12−14^ Each strategy has its merits as well as weak points, and the selection of the most appropriate one is a non-trivial task requiring considerable experience for their successful application. Recently, two computational approaches, FireProt^15,16^ and PROSS,^17,18^ have been developed to design multiple-point stabilizing mutations in an autonomous and user-friendly manner without the need for excessive experimental screening. Both approaches combine phylogenetic analysis and energy calculations using Rosetta^19^ to design stabilizing multiple-point mutations. Although both tools have been successfully applied to stabilize several structurally and functionally distinct proteins,^15,20^ the objective evaluation of their performance is difficult since the number of unsuccessful cases is unknown.

In this study, we compare several stabilization strategies, including rational design of disulfide bridges, *in silico* saturation mutagenesis, FireProt, and PROSS. We subjected individual methods to their application limits during the design of stabilizing mutations in a protein that has previously undergone intensive engineering – the stable haloalkane dehalogenase DhaA115.^15,21^ The main rationale behind our selection is twofold. First, DhaA115 displays activity toward a broad range of halogenated substrates, many of which are toxic and carcinogenic compounds persistent in the environment, e.g., 1,2-dichloroethane and 1,2-dibromoethane. This makes the enzyme particularly valuable for biotechnological applications that detoxify and degrade harmful pollutants.^22^ Stabilization allows the enzyme to operate under harsh conditions required by the bioindustry. Stabilized DhaA variants are valuable scaffolds for designing or improving activity toward other biotechnologically relevant halogenated substrates, e.g., 1,2,3-trichloropropane or yperite, due to increased tolerance to destabilizing mutations.^23,24^ Second, DhaA115 already demonstrates considerable stability with an apparent melting temperature of ∼73 °C. Its further stabilization is challenging due to the low probability of finding the mutations that would strengthen the previously optimized network of interactions. Although several studies achieved remarkable stabilization, most of them were carried out on mesophilic proteins with relatively low melting temperatures,^25^ involved stabilization of oligomeric interfaces,^26^ or extensive experimental screening.^27,28^ Here, we focus on designing multiple stabilizing mutations *in silico* rather than experimental screening of many single-point mutations and their subsequent combination. This strategy aligns with our long-term effort to develop a fully automated stabilization platform requiring minimal user intervention.

**Figure 1:**
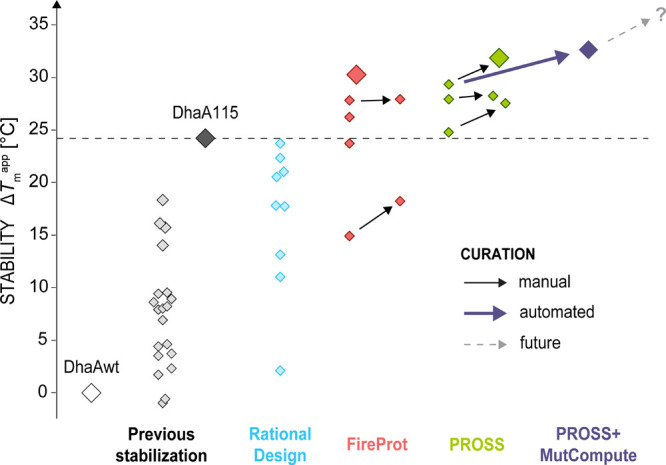
Overview of haloalkane dehalogenase DhaA stabilization. Wild-type enzyme DhaAwt (*T*_m_^app^ ∼ 50 °C) has been previously stabilized by 11 mutations designed by FireProt to yield DhaA115.^15^ In the current study, different strategies for further stabilization of DhaA115 (*T*_m_^app^ ∼ 73.3 °C, black) were tested to compare their performance, including rational design (blue), FireProt (red), and PROSS (green). The apparent melting temperatures of individual variants are depicted as diamonds. Some of the mutations designed by the automated stabilization tools were removed based on their manual (solid black arrows) or automatic (i.e., using MutCompute,^13^ solid violet arrow) curation, resulting in further stability increase. The gray dashed arrow and the question mark indicate the hidden potential of the automated approaches that can be unlocked by the implementation of automated curation protocols into the automated stabilization tools.

Compared to our rational design efforts, we observed a superior performance of the automated protocols FireProt and PROSS in successfully identifying multiple-point stabilizing mutations. Importantly, we show that manual curation of the predicted mutations further improves the stability of the designs, and we successfully test the machine-learning algorithm that shows promise for automation of this process in the future. We subjected the most stable designs to biophysical and biochemical characterization to quantify their stability and catalytic efficiency. Finally, we analyze the newly solved high-resolution structures of the most stable variants to reveal the structural basis of the mutational effects on protein stability. The 8.4 °C stabilization in terms of the apparent melting temperature compared to the template DhaA115 (overall stabilization over 30 °C compared to wt) and the simultaneous improvement of the catalytic properties of the best designs reflects the great potential of current automated stabilization platforms (Figure 1). We believe that our study shows a novel way to push the limits of these platforms and will help their improvement toward fully autonomous, accurate engineering tools of highly thermostable enzymes in the future.

## Results

### HDX-MS Analysis Reveals that the Cap Domain of DhaA115 Is Prone to Thermal Unfolding

**Figure 2:**
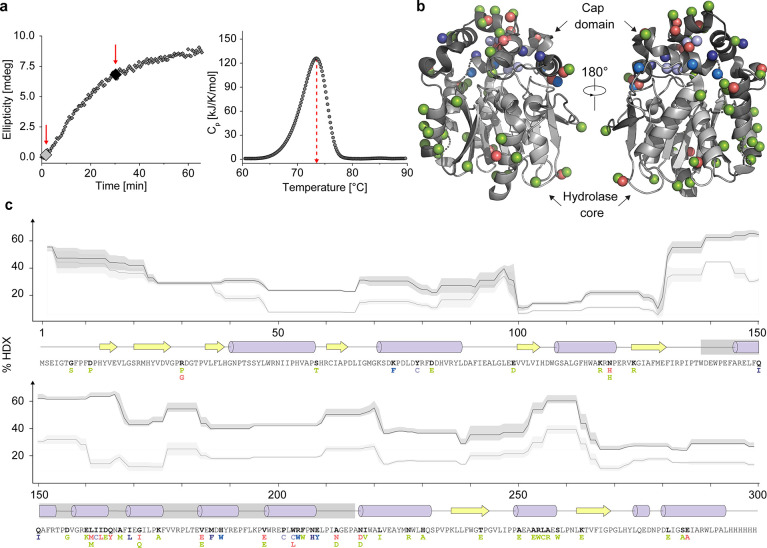
Biophysical analysis of DhaA115. (a) Thermal unfolding of DhaA115. Left: unfolding kinetics of DhaA115 monitored by CD spectroscopy at 67 °C for 1 h. The red arrows indicate time points where hydrogen/deuterium exchange profiles of native and denatured states (shown in panel c) were analyzed, respectively. Right: differential scanning calorimetry (DSC) scan of DhaA115 from 20 to 95 °C at 1 °C/min scan rate. The red dashed arrow indicates *T*_m_^app^. (b) The structural context of the DhaA115 residues that were mutated within this study. The DhaA115 backbone is color-coded according to the level of deuteration observed after 30 min at 67 °C from light gray (low) to black (high). The spheres correspond to the residues that were mutated based on the Disulfide by Design 2.0 (light blue), Rosetta ddg monomer (interdomain, marine blue; intradomain, dark blue), FireProt (red), and PROSS (green). (c) Thermal unfolding of DhaA115 monitored by hydrogen-deuterium exchange (HDX-MS). Top: hydrogen-deuterium exchange profile of native (light gray) and unfolded (dark gray) states of DhaA115 corresponding to the time points at 67 °C highlighted in (a). Middle: secondary structure of DhaA115 including β-strands (yellow arrows), α-helices (purple barrels), and loops (black lines). The cap domain is marked by the gray box. Bottom: amino acid sequence of DhaA115, including mutated residues color-coded according to (b).

The target protein used in this study is the computationally stabilized haloalkane dehalogenase DhaA115 designed previously using the web server FireProt.^15^ Eleven mutations that were originally introduced in the wild type increased its apparent melting temperature (*T*_m_^app^) to approx. 73 °C (Δ*T*_m_^app^ ∼ 23 °C, Figure 2a), making it the most stable haloalkane dehalogenase to date. Although the energy and structural basis of DhaA115 thermostability have been elucidated by the global analysis of thermal denaturation experiments and crystallographic analyses, respectively,^21,29^ the specific structural regions most prone to unfolding are currently unknown. To this end, we have carried out hydrogen/deuterium exchange coupled with mass spectrometry detection (HDX-MS) of DhaA115 after one- and thirty-minute incubation at 67 °C to compare solvent accessibility of residues in the native and the denatured states, respectively (Figure 2c). The most significant difference in deuteration between each conformation was observed in residues 133–237, mainly in the region spanning cap domain helices α4 to α7 (residues 144–187). This indicates that their solvent accessibility increases during the unfolding of the cap-domain. The results of HDX-MS are further supported by the decrease of ellipticity at 227 nm observed during the unfolding of DhaA115 at 67 °C by circular dichroism (CD) spectroscopy (Figure 2a), corresponding to the loss of helical structure. Based on these observations, we hypothesized that strengthening the interactions between the cap and core domains and within the cap domain could lead to stabilization of the DhaA115.

### Rationally Designed Mutations in the Cap Domain Failed To Stabilize DhaA115

Based on the visual inspection of the DhaA115 structure, we selected two specific interfaces at the most deuterated regions for targeted mutagenesis. The first region, the intra-domain interface, involves residues forming the interface between the cap-domain helices, whereas the second, inter-domain interface, is formed by the residues located between the cap-domain and the hydrolase core. The list of the residues forming the interfaces and the results of the *in silico* mutagenesis are provided in Supplementary Tables 1–3.

First, we introduced disulfide bridges between the least stable regions, creating a covalent staple that locks them together and stabilizes against unfolding. Using Disulfide by Design 2.0,^30^ we identified I162C + W203C and Y79C + P201C located in the intra-and inter-domain interfaces, respectively, as the most promising pairs in each region, and introduced them individually and in combination with DhaA115 (Figure 2). Unfortunately, the comparison of their thermal denaturation in reducing and non-reducing conditions revealed that the positive stabilization effect of the covalent cysteine bridges was countered by the destabilization caused by the mutations (Supplementary Figure 1). Apart from the lowered stability compared to DhaA115 (Table 1), their soluble expression was also significantly compromised.

**Table 1:** Overview of the Mutant Variants of DhaA115 Designed and Experimentally Characterized in This Study^a^

software	design	DhaA	mutations	*T*_m_^app^ [°C]
FireProt	template	115	none	73.3 ± 1.8
DbD	interdomain	181	Y79C, P201C	66.8 ± 0.0
	intradomain	182	I162C, W203C	72.8 ± 1.0
	combined	183	DhaA181 + DhaA182	71.4 ± 1.2
Rosetta	interdomain	184	K74F	62.5 ± 2.5
	interdomain	185	E208Y	66.9 ± 2.0
	interdomain	188	H188W, R204W	70.1 ± 2.7
	intradomain	186	Q150I, I169L	69.6 ± 2.0
	intradomain	187	Q150I, I169L, M186F, N207H	60.1 ± 2.9
	combined	189	Q150I, I169L, M186F, H188W, R204W, N207H	51.2 ± 0.1
FireProt	energy	211	Q165Y, G171I, A212N	75.3 ± 1.3
	ratio	212	R30G, V184E, V197E, N217D	76.9 ± 0.6
	ratio*	213	DhaA212-**R30G**	77.0 ± 0.7
	majority	214	N119H, L161M, I163L, W203L, E285A	64.0 ± 0.9
	majority*	215	DhaA214-**N119H, W203L, E285A**	67.3 ± 1.0
	combined	216	DhaA211 + DhaA213 + DhaA215	72.8 ± 0.9
	combined*	223	DhaA211 + DhaA213	79.3 ± 0.9
PROSS	design 2	217	D11P, R30P, N119H, K124R, V184E, V197E, N217D, N227R, H230A, T242E, S258W	77.0 ± 0.7
	design 2*	218	DhaA217-**R30P, N119H, H230A, S258W**	77.3 ± 1.5
	design 5	219	DhaA217 + D156G, F205W, A212D, I218V, L221I, R254W, K263E, S284R	73.8 ± 0.7
	design 5*	220	DhaA219-**F205W, R254W, K263E, S284R**	76.6 ± 1.8
	design 8	221	DhaA219 + G7S, S58T, D82E, E99D, K117R, Q150K, E160K, L161M, D164E, A167M, G171Q, K175A, A250E, A253E, L255C, A256R, L281E	78.4 ± 0.9
	design 8*	222	DhaA221-**R30P, N119H, H230A, S258W, F205W, R254W, K263E, S284R**	80.9 ± 0.7
PROSS, MutCompute		231	DhaA222-**D11P, A212D, and L221I**	81.7 ± 0.9

a The asterisk denotes variants in which mutations designed by the automated tools have been removed based on manual curation described in the text (bold). The values of *T*_m_^app^ are means and standard deviations obtained from three different techniques: DSC, CD, and differential scanning fluorimetry (DSF), except for variants containing disulfide bridges where the values represent means and standard deviations of *T*_m_^app^ from three replicates of DSF measurements.

As an alternative strategy, we carried out an *in silico* saturation mutagenesis of all the residues in the identified regions and evaluated the mutational effects using a combination of Rosetta^19^ and HotSpot Wizard^31^ (Supplementary Tables 2–3). Only the mutations that passed the thresholds of both software were selected and ranked according to their risk of being false positives based on the HotSpot Wizard mutability score. The two risky mutations were introduced individually, while the rest were combined into two double-point, one four-point, and one six-point mutant selected for experimental characterization (Table 1 and Supplementary Figure 2). Despite the predicted stabilization effects, none of the variants displayed increased thermostability. The two single point mutations (K74F and E208Y) lowered the *T*_m_^app^ of DhaA115 by 11 and 6.4 °C, respectively. Two pairs of mutations, Q150I + I169L and H188W + R204W, were only slightly destabilizing (Δ*T*_m_^app^ = −3.5 °C), while destabilization of the double-point mutant M186F + N207H was almost thrice as high (Δ*T*_m_^app^ = −9.5 °C). The combined six-point mutant significantly compromised the structural integrity of the respective variant, resulting in its poor expression and increased aggregation.

### Multiple-Point Mutations Designed by the Automated Computational Platforms FireProt and PROSS

Both our rational strategies failed to stabilize DhaA115. Arguably, the chances of finding a combination of stabilizing mutations by rational design of a protein with an already highly optimized network of stabilizing interactions are challenging. A difficult-to-engineer protein such as DhaA115 represents a suitable target for benchmarking two state-of-the-art automated hybrid stabilization web tools FireProt^15,16^ and PROSS.^17,18^ Both methods construct a multiple sequence alignment to find non-conserved positions suitable for mutagenesis and then use Rosetta to predict the free energy of stabilization (ΔΔ*G*) introduced by the different substitutions. However, they differ in the general pipeline and implemented tools, thresholds, and filters. FireProt also runs energy calculations with FoldX calculations before Rosetta, or just FoldX for evolution-based mutations. We employed both platforms to design potentially stabilizing mutations in DhaA115 to explore whether they can overcome the drawbacks experienced during the rational design.

FireProt involves evolutionary- and energy-based strategies to design stabilizing mutations.^15^ The evolutionary-based approach identifies positions where a wild-type amino acid differs from the most prevalent one in the multiple-sequence alignment of homologous proteins. The back-to-consensus (BTC) mutations were selected based on (i) the number of times the consensus residue appeared at this position in the alignment – the majority method or (ii) based on the ratio of its frequency in the alignment compared to the original residue – the ratio method. The energy-based design uses conservation and correlation analyses to filter the residues for *in silico* saturation mutagenesis, which are then evaluated by FoldX^32^ and Rosetta^19^ (Supplementary Table 4). One design from each method was selected, prepared, and experimentally characterized (Table 1, Supplementary Figure 3). The three energy-based mutations increased the *T*_m_^app^ of DhaA115 by 2 °C (DhaA211). Strikingly, the two sets of evolutionary-based mutations showed the opposite effects on the stability of DhaA115. The BTC mutations suggested by the ratio method increased *T*_m_^app^ by 3.6 °C (DhaA212), while the mutations selected by the majority method destabilized DhaA115 by 9 °C (DhaA214). Interestingly, all but two (R30G, E285A) mutations suggested by FireProt are localized within or near the cap-domain interfaces identified by the HDX-MS experiments (Figure 2c). This reassures us that the structural regions targeted in our previous stabilization efforts were identified correctly. However, their stabilization requires a more sophisticated and robust computational approach, such as the one used in FireProt.

Next, the automated web server PROSS^17,18^ was used to design stabilizing mutations in DhaA115. The mutations were selected by a two-step filtering process that includes reducing the sequence space using a position-specific scoring matrix (PSSM) derived from the alignment of homologous proteins, followed by Rosetta force-field-based calculations.^19^ In the final step, PROSS combined the most likely stabilizing mutations into 9 different designs (Supplementary Table 5). The number of mutations in the resulting variants ranged from 5 in the most stringent to 48 in the most permissive design based on the threshold values of the Rosetta energy scores. Following the developers’ recommendation, we have selected three designs containing 11 (DhaA217), 19 (DhaA219), and 36 (DhaA221) mutations for the experimental characterization (Table 1). Stability improvement was observed in all PROSS variants to various degrees, including weak (DhaA219, Δ*T*_m_^app^ = 0.5 °C), medium (DhaA217, Δ*T*_m_^app^ = 3.8 °C), and high (DhaA221, Δ*T*_m_^app^ = 5.1 °C). In contrast to FireProt and in line with the PROSS strategy, the designed mutations were distributed across the whole structure of DhaA115 (Figure 2b,c). Their large number makes the dissection of the stabilization effect tedious. However, since each design contains all the mutations from the previous one (e.g., DhaA219 = DhaA217 + additional mutations), some of the 8 mutations added to DhaA219 must be destabilizing. The stability restores and increases further by the additional 17 mutations in DhaA221. The latter is the most thermostable of the three and is 1.5 °C more stable than the best variant designed by FireProt (DhaA212). Considering that DhaA221 contains 3 mutations from DhaA212 (V184E, V197E, and N217D) and predicts a different mutation into position R30 (P instead of G), the marginal increase in stability is achieved by the additional 32 mutations, which make up 10% of the overall protein sequence. Altogether, PROSS identified 5 identical mutations also found by FireProt and 3 positions where the mutated residue differed between the two approaches (R30, G171, and A212). The mutations I163L, Q165Y, W203L, and E285A, suggested by FireProt, were not identified as stabilizing by PROSS.

### Identification of False Positive Mutations Further Improves Thermostability

FireProt and PROSS succeeded where we failed with the manual rational design and increased the thermostability of DhaA115 by 3.6 and 5.1 °C, respectively. Before proceeding to the experimental validation of these proposed designs, we additionally decided to inspect all suggested mutations in closer detail to reveal any potentially false positive predictions. Each mutation was evaluated by (i) its visual inspection within the structural context of DhaA115, (ii) Missense3D prediction tool for the identification of potentially disruptive mutations,^33^ and (iii) HotSpot Wizard analysis of the mutability of the residues. As a result, we have excluded several mutations designed by FireProt and PROSS and experimentally validated the resulting effects on the stability in parallel with the originally proposed designs described in the section above (Table 2, Supplementary Figure 4).

**Table 2:** Mutations Manually Eliminated from the FireProt and PROSS Designs^a^

mutation	web server	reasons for elimination	HSW score	MutCompute log(*P*)	Δ*T*_m_^app^ upon removal in (DhaA variant)
R30G/P	FireProt	lost H-bonds to D26 and G28	7	–7.3/–6.7	neutral (DhaA213/218)
	PROSS				
N119H	FireProt	lost H-bond to R122	4	–3.0	+3.4 °C (DhaA215)
	PROSS				
W203L	FireProt	buried to exposed hydrophobic residue	4	–9.0	
E285A	FireProt	lost H-bond to R288	6	–6.3	
H230A	PROSS	lost H-bonds to M226 and S258	6	–4.7	neutral* (DhaA218)
S258W	PROSS	lost electrostatic interactions with H230, R254, L255	6	–7.5	
F205W	PROSS	reduction of active site volume by 18.4 Å^3^	6	–1.0	+2.8/2.5 °C (DhaA220/222)
R254W	PROSS	lost electrostatic interaction with E251	2	–6.2	
K263E	PROSS	lost electrostatic interaction with E285, charge reversal	6	–3.7	
S284E	PROSS	lost H-bonds to D280 and L281	6	–4.2	

a The reasons for elimination are based on: (i) visual inspection in the DhaA115 structure, (ii) Missense3D, and (iii) HotSpot Wizard (HSW) analyses. HSW scores range from 1 to 10, from lowest to highest mutability. The MutCompute log probabilities of substitutions and the experimentally determined stability changes upon mutation (Δ*T*_m_^app^) are also presented with the respective variant in the brackets. Asterisks denote that the effect was observed when mutations H230A and S258W were removed together with R30P and N119H.

The mutation of W203 introduced during the rational design of disulfide bridges resulted in the destabilization of the respective variants in our first stabilization attempt (DhaA182 and DhaA183, Table 1). Arguably, removing this tryptophan alters the packing density or disrupts favorable cation–pi interactions with surrounding residues, e.g., R159. Indeed, the elimination of W203L from DhaA214, together with N119H and E285A, improved *T*_m_^app^ by 3 °C, indicating that our concerns about mutations of this residue were justified. However, the stability of the remaining double-point mutant (DhaA215) was still below DhaA115 (Δ*T*_m_^app^ = −5.9 °C, Table 1). In contrast, removing the putatively destabilizing mutation R30G from DhaA212 variant (DhaA213) had a negligible effect on its stability (Δ*T*_m_^app^ = 0.1 °C, Table 1). Based on our detailed inspection of the multiple sequence alignment, we speculate that a salt bridge is formed between D26 and R60, rather than R30. Specifically, we observed that (i) other amino acids that do not form salt bridges are frequently found in position 30, (ii) R60 is highly conserved, and (iii) there is a high frequency of negatively charged amino acids D or E in position 26 (Supplementary Figure 5). All FireProt mutations deemed “safe” based on our curation showed an additive effect, judged by the comparison of stabilities between the combined variant DhaA216 and DhaA115. Following the experimental validation of all FireProt designs, we removed the destabilizing mutations L161M, I163L from DhaA216 and obtained the most stable FireProt variant, DhaA223, with 6 °C higher *T*_m_^app^ compared to DhaA115 (Table 1).

The large number of mutations predicted by PROSS makes the dissection of their individual contributions to the overall protein stability difficult. We have identified 8 out of the 36 mutations as potentially destabilizing before proceeding to experimental validation (Table 2 and Supplementary Figure 4). The experiments revealed that the removal of 4 mutations (R30P, N119H, H230A, and S258W) from the DhaA217 had only a marginal effect on the stability (DhaA218, Δ*T*_m_^app^ = 0.3 °C, Table 1), indicating that they were mostly neutral. In contrast, further removal of F205W, R254W, K263E, and S284R from both DhaA219 and DhaA220 increased their stability by ∼3 °C (DhaA220 and 222, Table 1), suggesting that these were indeed destabilizing. Strikingly, none of the mutations removed from FireProt and PROSS designs showed coevolutionary signals with the putative interacting residues suspected by us, suggesting either a lack of their conservation within the protein family or some other mechanism of destabilization (Supplementary Tables 6 and 7).

The manual curation of the mutations designed by both FireProt and PROSS, supported by analyses by Missense 3D and HotSpot Wizard, improved the stability of the original variants by approximately 3 °C, resulting in considerable thermostable proteins with *T*_m_^app^ of 79 and 81 °C, respectively. However, we acknowledge that our identification of potentially false positive predictions has its limitations. For example, mutations L161M and I163L designed based on the BTC-majority approach of FireProt were destabilizing DhaA115 (DhaA215, Δ*T*_m_^app^ = −6 °C, Table 1). The removal of L161M from DhaA222 could thus further improve its stability. However, such extrapolation goes against the philosophy of these automated platforms, which strive for minimal user interference. Based on this experience, we speculated whether a context-dependent computational curation of the mutations designed by FireProt or PROSS could bypass the subjective manual evaluation and improve the accuracy of these web servers. As a proof-of-concept, we analyzed the excluded mutations by the structure-based deep learning algorithm MutCompute,^13^ which was recently employed to stabilize DNA polymerase^34^ or PETase.^25^ The algorithm generates a probability score for each possible substitution of the input protein based on the chances of finding such substitution in the given structural context within its training dataset.^13^ MutCompute correctly predicted that the template residues are preferred over the mutations proposed by FireProt and PROSS for all experimentally confirmed destabilizing mutations (Table 2). It also suggested that L161M and I163L are unlikely to stabilize DhaA115 (Supplementary Table 8, Supplementary File 7). Moreover, the removal of 3 mutations possessing a low probability of substitution (D11P, A212D, and L221I) from DhaA222 resulted in a further increase of thermostability by 1 °C (DhaA231, Table 1, Supplementary Figure 3). These encouraging results suggest further improved prediction accuracy by complementing phylogenetic analysis and force-field calculations with additional machine learning-based or other filtering protocols (Figure 1).

Having succeeded in pushing the limits of haloalkane dehalogenase thermostability to a higher level, we next focused on advanced analysis of the mutational effects in the three most stable variants to gain deeper insights into the structure-function relationship of HLDs. First, we investigated the changes in protein energetics using the global analysis of protein denaturation. Next, we carried out a crystallographic analysis to elucidate the structural basis of the stabilization. Finally, we performed rigorous biochemical characterization to investigate their catalytical properties.

### Computationally Designed Mutations Stabilize the Native State and Increase Kinetic Stability

**Figure 3:**
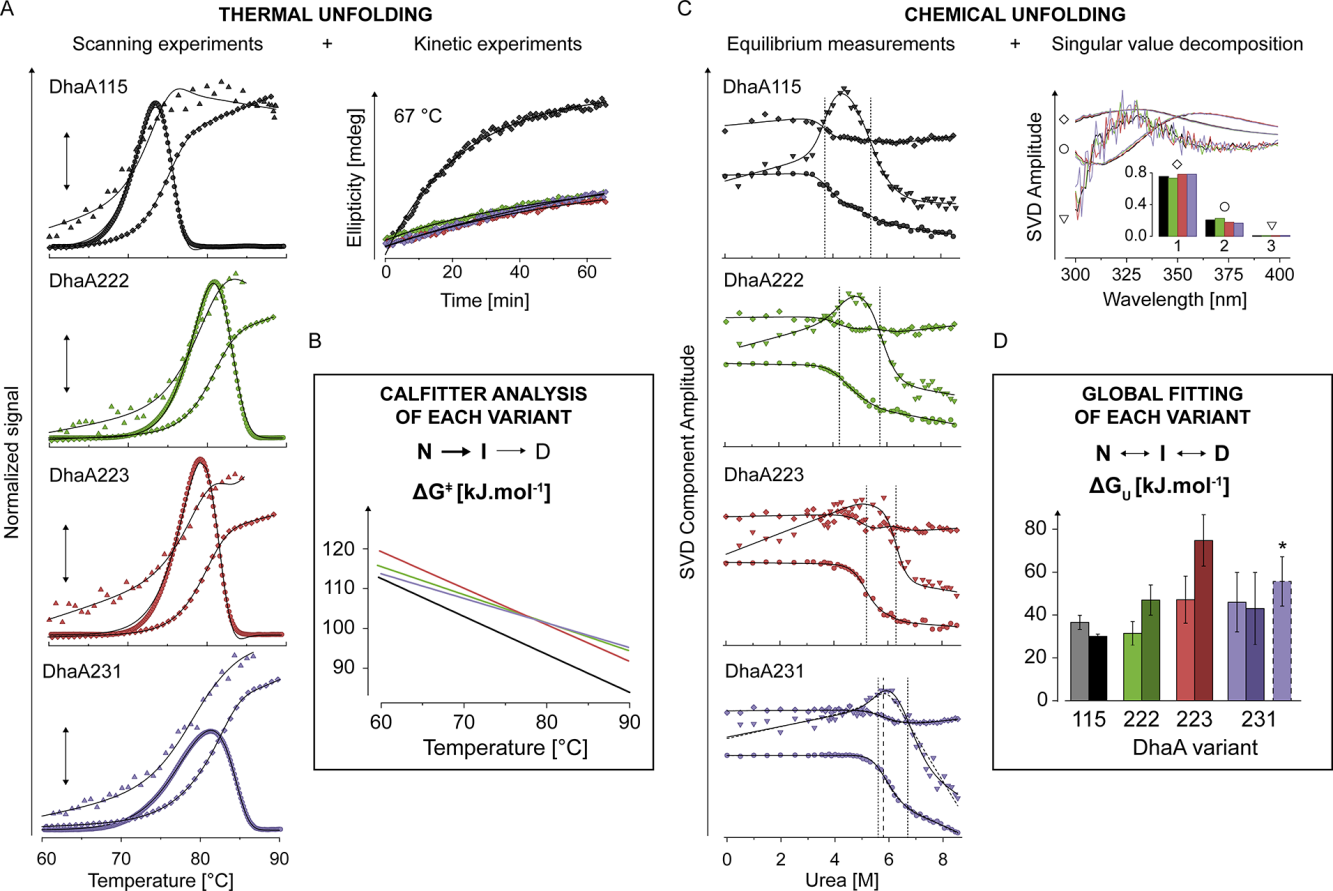
Comparison of stability between DhaA115 and the most stable variants DhaA222, DhaA223, and DhaA231. (a) Global fitting of thermal unfolding. The excess heat capacity (circles), ellipticity at 224 nm (triangles), and redshift of fluorescence spectrum (i.e., barycentric mean of fluorescence BCM, diamonds) scans at three different scan rates (0.5; 1; and 2 °C/min, 1 °C/min shown) were fitted globally together with the unfolding kinetics traces at four different temperatures (67 °C shown) to the two-step irreversible unfolding model using CalFitter.^36^ The fits of data for all variants are shown as black lines. Spectroscopic scans are shown as normalized values. The four graphs share the heat capacity scale indicated by the double-sided arrow in the middle. (b) Gibbs activation energy of unfolding. The energy barrier of the first unfolding step quantifies the kinetic stability of all variants. The Δ*C*_p_ was fixed to zero during the analysis. yielding a linear temperature dependence of the energy barriers. The linearity approximation holds reasonable truth only in the temperature range of unfolding shown in the graph. (c) Urea-induced denaturation. The four plots show singular value decomposition (SVD) components of fluorescence spectra change with urea concentration. The first three most significant SVD components are depicted by diamond, circle, and triangle, respectively, together with the global fits to the three-state unfolding model with native (N), intermediate (I), and denatured (D) states (solid black lines). Their basis spectra together with a bar graph of normalized singular values are shown in the top right corner of the figure. Fit of the DhaA231 denaturation to the simpler, two-state unfolding model is shown as dashed lines. The vertical dotted lines indicate the midpoints of unfolding transitions (*C*_m_). (d) Thermodynamic stability of DhaA115, DhaA222, DhaA223, and DhaA231. The bar graph depicts the Gibbs free energy difference between the native and intermediate states (Δ*G*_NI_, light colors) and the Gibbs free energy change between the intermediate and denatured states (Δ*G*_ID_, dark colors). The asterisk and purple bar with a dashed line indicate the Δ*G*_ND_ value derived from fitting the DhaA231 data to the two-state model. The black error bars represent standard errors of the fitting parameters.

The training of a novel generation of computational tools for protein stabilization largely depends on acquiring high-quality experimental data from protein stability screening.^35^ To quantify the energetic effects of the mutations beyond apparent melting temperatures and to obtain mechanistic insights into the extraordinary thermostability of DhaA222, DhaA223, and DhaA231 variants, we have probed their denaturation by multiple experimental techniques and analyzed the resulting data globally using the CalFitter web server.^36^ Similarly to DhaA115, the unfolding of both variants is characterized by a single endothermic calorimetric peak, redshift of the fluorescence spectra, and a decrease of ellipticity at 224 nm (Figure 3a). Their unfolding transitions are scan rate-dependent and irreversible beyond *T*_m_^app^ with no signs of aggregation based on the scattering, indicating kinetically controlled denaturation. Indeed, datasets of each protein fitted well to the two-step irreversible unfolding model (Figure 3a), allowing us to confidently quantify the stability of each variant in terms of the energy barrier of the first transition (Δ*G*^⧧^, Figure 3b). In the temperature range of the unfolding transitions (70–90 °C), the energy barrier of all three stable variants increased on average by 7–8 kJ/mol compared to the DhaA115 (Figure 3b, Supplementary Table 9). Specifically, this translates to approx. 7-, 12-, and 5-times longer half-lives at 70 °C of DhaA222, 223, and 231, respectively, compared to the 8-minute half-life of DhaA115 at this temperature. Since we simplified the analysis by not considering the heat capacity difference between the native and the unfolded states (Δ*C*_p_), the extrapolation of the Δ*G*^⧧^ values to lower temperatures is unreliable. Interestingly, the calorimetric enthalpies (Δ*H*_cal_) of DhaA222 and 223 increased by approximately 140 and 200 kJ/mol, respectively, but only by 50 kJ/mol in the case of DhaA231. These results indicate that although the newly designed mutations increased the energy barrier of unfolding of each DhaA variant, the underlying molecular mechanisms of the stabilization may differ between them.

In contrast to the thermal denaturation, all four proteins were almost completely and reversibly unfolded by high concentrations of urea, which allowed us to probe their thermodynamic stability at 25 °C (Figure 3c). We resolved the complex urea-induced effects on the protein intrinsic fluorescence spectra (including bidirectional change of the signal intensity and redshift of the spectral maximum) using the SVD (Figure 3c).^37^ The three most significant SVD components well represented all spectral datasets; thus, changes in their amplitudes were used for fitting. The resistance to urea-induced denaturation increased in the order of DhaA115 < DhaA222 < DhaA223 < DhaA231 and the best fit in all four cases was achieved using the three-state model of unfolding (Figure 3c, black lines). However, we observed considerable cross-correlation between the *m*-values and baseline parameters of the (mainly) intermediate state (where possible, we fixed the baseline parameters of native and denatured states during fitting). Consequently, the only statistically significant parameters for comparing the differences between the designed variants’ stabilities were the transitions’ midpoints (*C*_m_, dotted lines in Figure 3c, Supplementary Table 10). Interestingly, the extent of this correlation increased in the same order as described earlier due to the diminishing resolution of the individual transitions. This was most prominent in the unfolding of DhaA231, which fitted reasonably well to the two-state unfolding model (Figure 3c, dashed lines). Altogether, the trend indicates that stabilization of the selected DhaA variants simultaneously increases the cooperativity of unfolding, most likely by destabilizing the intermediate state.

Based on our mechanistic analyses, we can thus conclude that the newly introduced mutations increased both kinetic and thermodynamic stabilities of all three DhaA variants. The increased cooperativity of unfolding is an important observation as we hypothesize that the unfolding intermediates observed in other HLDs are responsible for aggregation. The new stabilized variants can shed light on a structure-stability relationship of HLDs in a follow-up study.

### Structural Analyses of DhaA223 and DhaA231 Reveal Networks of Stabilizing Interactions

We attempted to crystallize the most stabilized enzyme variants to provide structural insights into thermostabilization and computational design. We obtained diffraction-quality crystals of DhaA223 and DhaA231 enzymes that belonged to the space groups *P*12_1_1 and *P*2_1_2_1_2_1_ and diffracted at 1.5 and 1.3 Å resolution, respectively. The final models showed good values for deviation from the ideal geometries (Supplementary Table 11). Almost all the residues were built in densities, except for a few residues at the unstructured amino- and carboxy-terminal ends.

**Figure 4:**
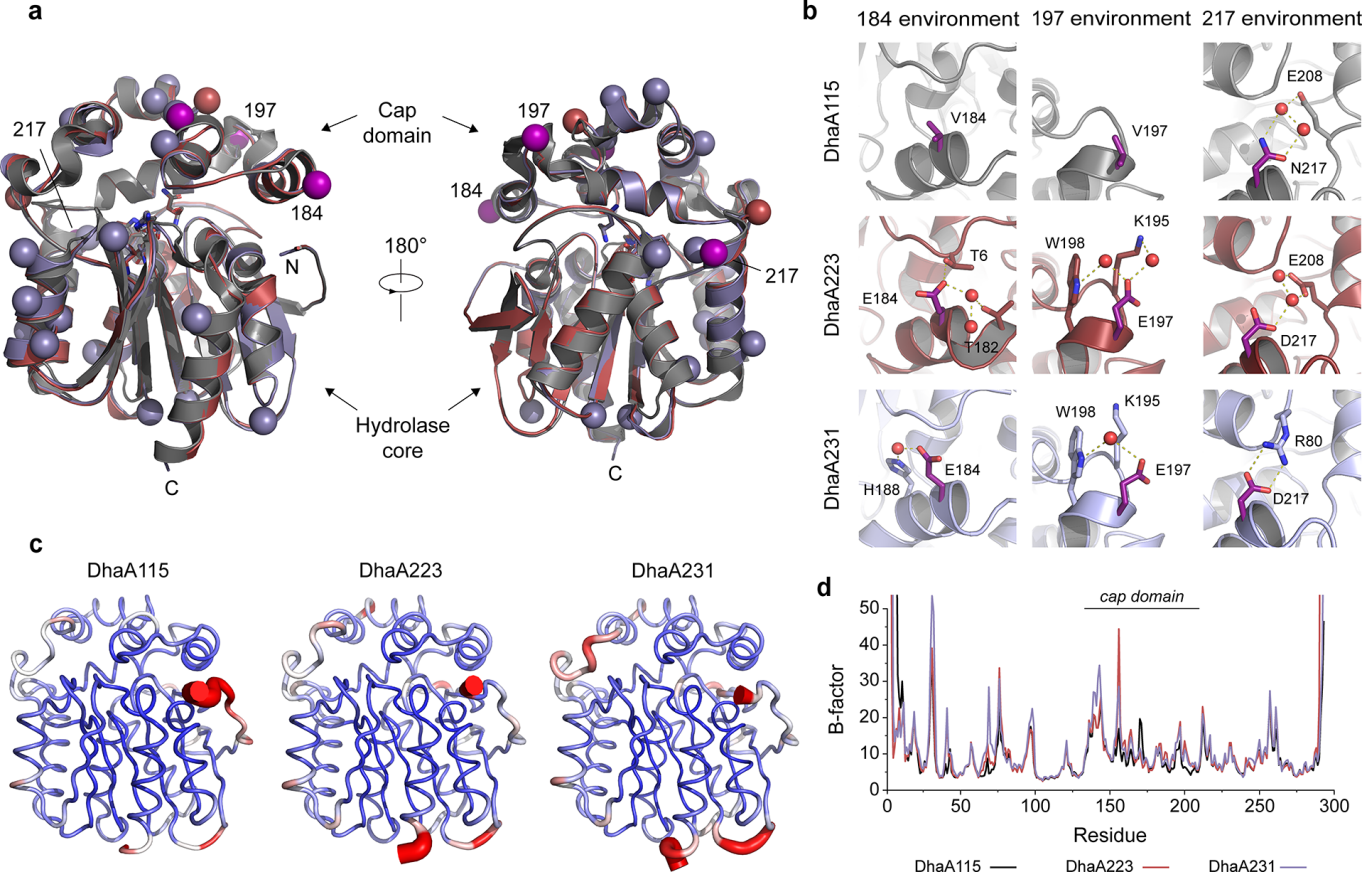
Crystal structures of DhaA223 and DhaA231. (a) Cartoon representations of DhaA223 (8OE2, red) and DhaA231 (PDB ID: 8OE6, dark blue) crystal structures aligned to the DhaA115 (PDB ID: 6SP5, gray). Residues of the catalytic pentad are shown as sticks. Stabilizing mutations are shown as spheres (pink spheres indicate mutations suggested by both FireProt and PROSS). (b) The structural context of selected stabilizing mutations. Newly formed stabilizing interactions involving other residues or water molecules (red spheres) are depicted by yellow dashed lines. (c) Projection of the MD-derived B-factors onto the structure of the DhaA variants. B-factors are normalized to the range of values between 0 and 40 and color-coded from the most rigid (blue) to the most flexible (red). (d) B-factors derived from the MD simulations were calculated based on the atomic fluctuations of the backbone atoms of the respective residues.

Both DhaA223 and DhaA231 enzymes adopt a canonical HLD fold like the template DhaA115 structure,^21^ making a compact αβα-sandwich architecture with a characteristic helical cap domain (Figure 4a). The positioning of catalytic pentad residues was not disturbed, which is a prerequisite for efficient catalysis. Importantly, a comparison of crystallographic structures showed that this round of stabilization is not accompanied by large backbone re-arrangements that were previously observed during the engineering of DhaA115 (Figure 4a). Here, we show that the protein backbones of DhaA223 and DhaA231 superpose well with that of the DhaA115, although some minor changes are observed in the hinge regions between the helical cap domain and the hydrolase core (Figure 4a, Supplementary Table 12). The crystal structures of DhaA223 and DhaA231 thus suggest that stabilization was achieved via strengthening intricate networks of residue-to-residue interactions, stabilizing the αβα-sandwich architecture. Moreover, multiple residues with polar or charged side chains were placed on the protein surface, which might also reinforce the stability of the designed proteins through new protein-solvent interactions. A notable example includes residues 184 and 197, where mutation of hydrophobic valines to glutamic acids created local hydrogen-bond networks involving water molecules (Figure 4b). Interestingly, analysis of B-factors derived from the MD simulations revealed that stabilization was not accompanied by increased proteins’ rigidity (Figure 4c,d). On average, both stabilized variants were even slightly more flexible than DhaA115. This implies that the increased stability is not a consequence of cap domain rigidification (Figure 4c,d) but that other forces, such as entropy and solvent effects, may play an important role. These are currently very difficult to predict, as demonstrated by some of our failed attempts described here, and represent an important challenge for improving force-fields.

### Stabilized Variants Exhibit Enhanced Catalytic Efficiency across a Broad Temperature Range

**Figure 5:**
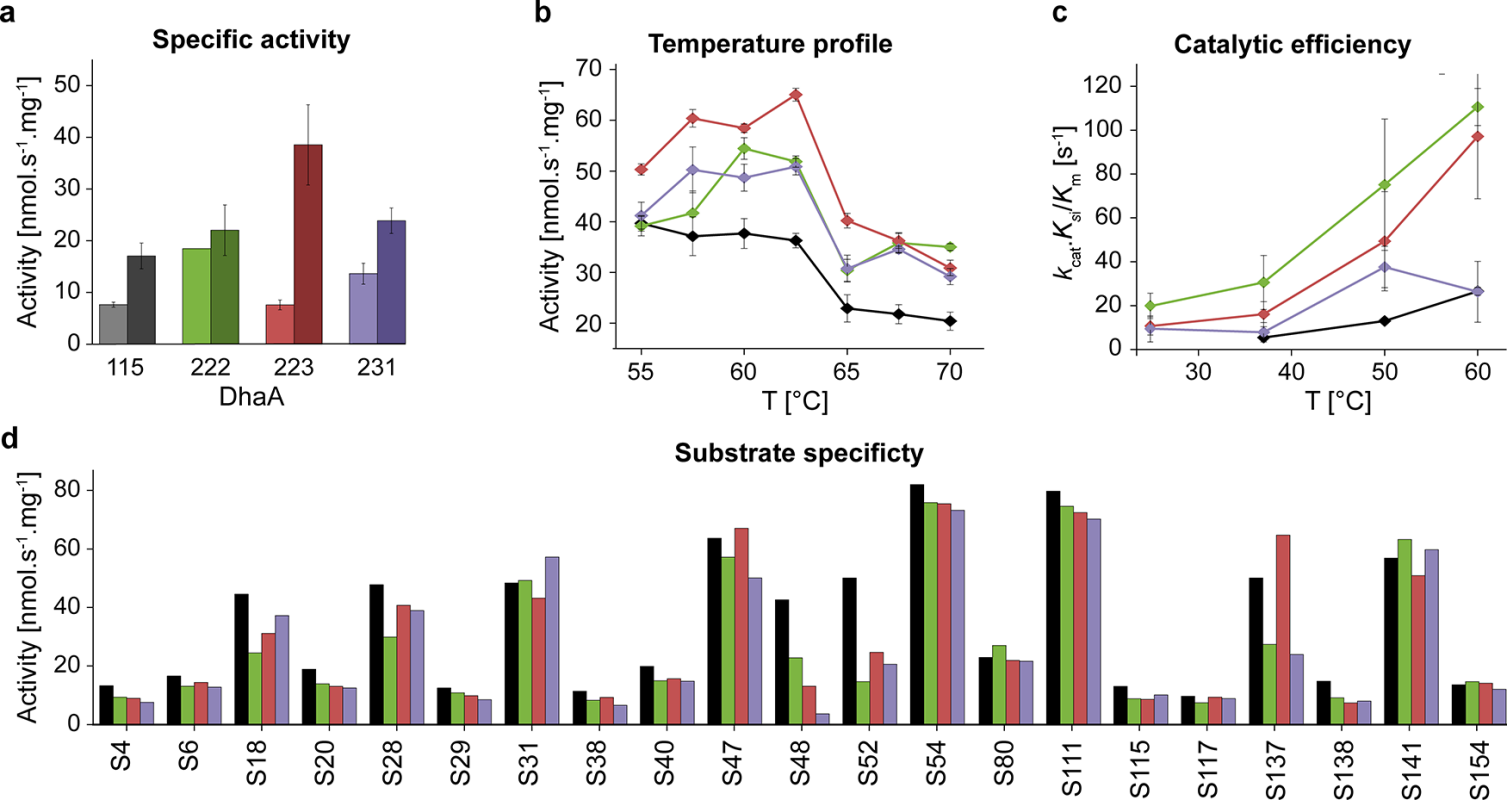
Catalytic properties of selected DhaA variants. DhaA115 (black), DhaA222 (green), DhaA223 (red), and DhaA231 (violet). (a) The specific activity of the selected variants toward 1,2-dibromoethane (DBE) was measured by the conventional colorimetric assay (100 mM glycine pH 8.6) at 37 (light bars) and 70 °C (dark bars). (b) Temperature activity profiles of the selected variants measured with DBE as the substrate by the pH-based assay (1 mM HEPES, pH 8.2) using MicroPEX.^38,39^ The error bars in (a) and (b) represent the standard deviations of the three independent measurements. (c) The catalytic efficiency of the selected variants adjusted for substrate inhibition (*k*_cat_·*K*_si_/*K*_m_) as a function of temperature. The error bars represent standard errors of the fitting parameters. (d) Substrate specificity profiles of the selected variants. The bars represent mean activity toward 21 halogenated substrates (see Supplementary Table 14 for the list of names) measured by the pH-based assay (1 mM HEPES, pH 8.2) at 60 °C using MicroPEX.^38,39^

To verify that the mutations did not compromise enzymatic function, we measured the dehalogenation activities of DhaA115, DhaA222, DhaA223, and DhaA231 with 1,2-dibromoethane (DBE) at 37 °C and 70 °C using a conventional colorimetric assay (Figure 5a). At 37 °C, the specific activity of DhaA222 (18 nmol·s^–1^·mg^–1^) was twice higher compared to DhaA115 and 223 (both ∼8 nmol·s^–1^·mg^–1^), closely followed by the activity of DhaA231 (14 nmol·s^–1^·mg^–1^) (Figure 5a, light bars). Interestingly, a significant increase of activity was observed for DhaA223 (39 nmol·s^–1^·mg^–1^) at 70 °C compared to only moderate enhancements of DhaA115, DhaA222, and DhaA223 activities: 17, 22, and 23 nmol·s^–1^·mg^–1^, respectively (Figure 5a, dark bars). The relatively low activity of DhaA115 at 70 °C can be attributed to its lower stability and partial denaturation at this temperature. In contrast, the difference in the activities of the new variants indicates different temperature dependencies of their catalytic properties. To investigate the enzyme activity in more detail, we employed a recently developed microfluidic platform MicroPEX^38,39^ to measure the temperature profile of dehalogenation activities (Figure 5b). The activities of the new variants toward DBE increased steadily from 55 to 62 °C, in contrast to the dehalogenation activity of DhaA115, which slowly decreased at this range. The sharp drop in the activities above 62 °C was caused by the instability of the pH-based assay used in the MicroPEX setup. Consequently, the difference in the temperatures of maximum activity (*T*_max_) between the three new variants and DhaA115 (∼5 °C) is arguably underestimated. Although the activities obtained by the conventional and microfluidic techniques differ in their absolute values due to the different experimental conditions, the results correlate with each other in the observed trends and confirm that the newly stabilized variants have higher activities toward DBE across a wider temperature range (Figure 5a,b, Supplementary Table 13).

Finally, we employed MicroPEX to measure the specific activities of all stabilized variants toward a panel of 27 standard halogenated substrates at 60 °C (Figure 5d). We observed that their substrate specificity profiles did not change compared to the DhaA115 template, showing activity toward 21 of them. Unfortunately, the remaining 6 substrates [1,2-dichloroethane (S37), 1,2-dichloropropane (S67), 1,2-dibromopropane (S72), (bromomethyl)cyclohexane (S119), 1,2-dibromo-3-chloropropane (S155), and 3-chloro-2-methylpropene (S209)] were not compatible with the MicroPEX platform, and therefore, their dehalogenation by the selected variants could not be measured. The enzymes are most active with iodinated and brominated substrates, including 1,3-diiodopropane (S54), bis(2-chloroethyl)ether (S111), 1-iodohexane (S31), bromobutyronitrile (S141), and DBE (S47). Importantly, all DhaA variants covert 1,2,3-trichloropropane (S80), whose degradation is highly desirable due to its high environmental toxicity and persistence.^40^

To retrieve insights into the catalytic mechanisms of the new variants, we carried out steady-state kinetic measurements at temperatures between 25 and 60 °C using isothermal titration calorimetry (ITC) and DBE as the substrate. Similar to DhaA115, the kinetics of DBE conversion by the newly designed variants showed clear signs of substrate inhibition,^29^ thus, we accounted for this during the fitting (Supplementary Figure 6). The thermodynamic signatures of catalysis differed between the FireProt-based variants (DhaA115 and DhaA223) and the PROSS-based ones (DhaA222 and DhaA231). The former ones show a moderate increase in turnover (eight- and five-fold in the case of DhaA115 and DhaA223, respectively) and lowered affinity toward substrate as indicated by the slight increase of both *K*_m_ and *K*_si_ (Supplementary Table 15) with the temperature increase from 37 to 60 °C. In contrast, the DhaA222 and DhaA231 have significantly increased turnover compared to the DhaA115 and 223 across the whole temperature range, reaching maxima of 29 and 21 s^–1^, respectively, at 60 °C (Table 3, Supplementary Table 15). This is accompanied by increased *K*_m_ values and weak temperature dependency of the substrate inhibition (Supplementary Table 15). Consequently, DhaA222 and DhaA223 reach similar levels of catalytic efficiency at 60 °C when adjusted for the substrate inhibition (i.e., *k*_cat_·*K*_si_/*K*_m_), which is 3–4 times higher compared to DhaA115 and DhaA231 (Figure 5c, Supplementary Table 15).

**Table 3:** Thermodynamic Analysis of DBE Conversion by the Selected Variants^a^

protein	*k*_cat_ [s^–1^]	*K*_m_ [mM]	*K*_si_ [mM]	*k*_cat_/*K*_m_ [s^–1^ mM^–1^]
**DhaA115**	**2.26 ± 0.04**	**0.070 ± 0.003**	**0.82 ± 0.04**	**32.4 ± 1.5**
Δ*G* [kJ·mol^–1^]	80 ± 3	–7 ± 1	–1 ± 4	
Δ*H* [kJ·mol^–1^]	76 ± 2	–32 ± 1	–13 ± 3	
–*T*Δ*S* [kJ·mol^–1^]	4 ± 2	24 ± 1	12 ± 3	
**DhaA222**	**28.9 ± 0.6**	**0.104 ± 0.007**	**0.40 ± 0.01**	**277.8 ± 19.8**
Δ*G* [kJ·mol^–1^]	73 ± 4	–6 ± 2	–2 ± 5	
Δ*H* [kJ·mol^–1^]	54 ± 3	–19 ± 2	–5 ± 4	
–*T*Δ*S* [kJ·mol^–1^]	19 ± 3	13 ± 2	3 ± 4	
**DhaA223**	**4.6 ± 0.4**	**0.067 ± 0.009**	**1.4 ± 0.4**	**69.3 ± 10.5**
Δ*G* [kJ·mol^–1^]	78 ± 6	–8 ± 4	1 ± 8	
Δ*H* [kJ·mol^–1^]	54 ± 6	–19 ± 4	–16 ± 7	
–*T*Δ*S* [kJ·mol^–1^]	25 ± 6	11 ± 4	17 ± 7	
**DhaA231**	**20.9 ± 4.8**	**0.24 ± 0.09**	**0.3 ± 0.1**	**86.1 ± 38.8**
Δ*G* [kJ·mol^–1^]	74 ± 7	–4 ± 13	–3 ± 13	
Δ*H* [kJ·mol^–1^]	59 ± 6	–29 ± 13	0 ± 12	
–*T*Δ*S* [kJ·mol^–1^]	15 ± 7	24 ± 13	–3 ± 12	

a Temperature dependencies of the turnover (*k*_cat_) and equilibrium constants (*K*_m_ and *K*_si_) were analyzed by Eyring’s and van’t Hoff’s plot, respectively, to yield change of Gibbs free energy (Δ*G*), enthalpy (Δ*H*), and entropy (Δ*S*) between the reactants and products (for *K*_m_ and *K*_si_), or the corresponding activation parameters between reactants and the transition state of the reaction (i.e., Δ*G*^⧧^, Δ*H*^⧧^, Δ*S*^⧧^ for *k*_cat_). The *k*_cat_, *K*_m_, *K*_si_, and *k*_cat_/*K*_m_ values determined at 60 °C are denoted in bold.

The thermodynamic analysis provides valuable insights into the observed catalytic effects (Table 3, Supplementary Figure 7). The increased turnovers of DhaA222 and DhaA231 stem from a decrease in the transition state’s energy barrier (7 and 6 kJ/mol, respectively). This change is driven mainly by reducing the enthalpic component (∼ΔΔ*H*^⧧^ kJ/mol, Table 3). The substrate inhibition of DhaA222 and DhaA231 is exergonic at the whole temperature range, in contrast to DhaA223, for which it becomes energetically unfavorable at high temperatures (Table 3). The substrate inhibition explains the differences between the specific activities of the DhaA variants at 70 °C, measured at saturating concentrations of DBE.

We conclude that the adjusted catalytic efficiencies of DhaA222 and DhaA223 variants are significantly enhanced compared to the DhaA115 across the broad temperature range. The primary goal for biocatalyst stabilization is to improve its operational efficiency at elevated temperatures. Our significant finding is that at the reference temperature of 60 °C, the newly constructed variants are stable for long-term operation and exhibit significantly higher catalytic activity compared to the DhaA115 template (Figure 5c, Table 3). The increase in catalytic efficiency is an intriguing secondary benefit obtained through the further stabilization of DhaA.

## Discussion

In this study, we carried out extensive testing of several stabilization strategies, including (i) rational design of disulfide bridges, (ii) stabilization of the structural weak spots, (iii) fully automated platform FireProt, and (iv) automated web platform PROSS. Our attempts to introduce new intra- and inter-domain contacts by designing disulfide bridges or adding stabilizing substitutions by *in silico* saturation mutagenesis were unsuccessful even though we have assessed the mutability by HotSpot Wizard^31^ and designed mutations using the Rosetta ddG calculations.^19^ Arguably, the *talaris2014* force-field used by Rosetta^41^ may overestimate the hydrophobic interactions and underestimate the electrostatic contributions, e.g., for single-point mutations K74F and E208Y. Moreover, the protein–water interactions are neglected since the algorithm uses an implicit term for the solvent. This may be crucial since it has been shown that the water-mediated hydrogen bonds and electrostatic networks are pivotal for the stability of many proteins, including DhaA115^21^ and its stabilized variants described here. Optimization of the electrostatic term and implementing a more robust approach to the protein–water interactions could, therefore, be considered in future studies.

The successful protein stabilization by FireProt and PROSS highlights the importance of implementing phylogenetic constraints to narrow the sequence space. FireProt removes correlated mutations and favors evolutionary-conserved modifications from back-to-consensus analysis, whereas PROSS uses the evolutionary information to eliminate rarely observed mutations.^16,17^ Consequently, the number of proposed substitutions is approximately four times higher in the variants designed by the PROSS (11–36 mutations) than those predicted by the FireProt (3–8 mutations). The fact that 32 additional mutations in DhaA222 make the protein only marginally more stable compared to DhaA223 suggests that most of them are neutral or their effects are mutually compensated. Some level of compensation might also occur for FireProt-based mutations, albeit with lower probability due to their small number. In our case, eliminating potentially disruptive mutations from these designs further increases apparent melting temperatures by 2–3 °C. The manual selection of these mutations is admittedly case-dependent. Here, we use the convolutional neural network MutCompute to bypass the subjective evaluation of mutations and improve their filtering. The analysis supported our conclusions about the disruptive mutations and, more importantly, succeeded in identifying three additional false positives, as demonstrated by the improved stability of DhaA231. Incorporating MutCompute or another similar algorithm into the automated web servers could improve their accuracy and enhance their performance in identifying multiple-stabilizing mutations.

Our study yielded three newly engineered haloalkane dehalogenases that demonstrated considerable improvements in stability. We show that the mutations designed by FireProt and PROSS stabilized the native state of DhaA115 and increased the unfolding energy barrier, shifting their maximal activity temperatures above 65 °C. We also observe that the stabilization of DhaA is accompanied by the destabilization of an intermediate state based on the increasing cooperativity of unfolding (most noticeably for DhaA231). Structural analysis of the newly solved structures of DhaA223 and DhaA231 revealed a strengthened network of stabilizing interactions induced by the mutations. We found that the newly introduced mutations form new interaction networks, including water molecules, which further underlies the need for developing effective computational tools for modeling protein-solvent interactions.

The improved kinetic stability allowed the enzymes to operate at temperatures above 65 °C on 10 times longer timescales than the template DhaA115. The substrate specificity remained mostly unchanged, and all new variants showed considerable activity. Remarkably, the catalytic efficiency exhibited a notable enhancement, specifically targeting difficult-to-convert industrial side product 1,2,3-trichloropropane and toxic environmental pollutants, e.g., 1,2-dibromoethane.^42^ Their catalytic efficiency toward the latter was improved across a broad temperature range (25–60 °C), reaching the highest observed catalytic efficiencies observed for this family of enzymes. The thermodynamic analysis confirmed that the enhanced catalytic properties are related to the mutations and cannot be attributed solely to the Arrhenius effect or faster diffusion and elevated substrate solubility at higher temperatures. The mutations globally increased the turnover by decreasing the activation enthalpy of the chemical reaction for the substrate 1,2-dibromoethane. The maximal catalytic efficiencies of the best variants, adjusted for substrate inhibition, are three to four times higher than DhaA115 and comparable to the most active naturally occurring or computationally designed dehalogenases to date.^43^

## Conclusions

Our results show that protein stabilization by the automated hybrid stabilization frameworks FireProt and PROSS is a highly potent strategy for designing stable and catalytically efficient enzymes. We further demonstrate that manual curation of multiple point mutations could remove false positive predictions and enhance protein stability. Additionally, we showed promising results for future automation of this process that could replace manual curation and improve predictions without extensive expert knowledge. Finally, our study yielded three highly thermostable haloalkane dehalogenases with excellent application potential in the bioindustry.

## Supporting Information Available

Detailed description of the *in silico* and experimental methodology used in this work (PDF)
Supplementary figures and tables (PDF)
Results of the global analysis of DhaA115 temperature denaturation (XLSX)
Results of the global analysis of DhaA222 temperature denaturation (XLSX)
Results of the global analysis of DhaA223 temperature denaturation (XLSX)
Results of the global analysis of DhaA231 temperature denaturation (XLSX)
Results of MutCompute analysis of DhaA115 (XLSX)
