# Supplementary material for: Advancing Enzyme’s Stability and Catalytic Efficiency through Synergy of Force-Field Calculations, Evolutionary Analysis, and Machine Learning
Source: ACS Catal. 2023 Sep 11;13(19):12506–18. doi: 10.1021/acscatal.3c02575 (PMC10563018; doi:10.1021/acscatal.3c02575)
Supplement: Supplementary file 6 — cs3c02575_si_006.pdf [file cs3c02575_si_006.pdf]

SUPPLEMENTARY FIGURES AND TABLES

Advancing Enzyme´s Stability and Catalytic Efficiency through Synergy of Force-Field Calculations, Evolutionary Analysis and Machine Learning

Antonin Kunka[1,2]*‡†, Sergio M. Marques*[1,2]*‡, Martin Havlasek*[1]*, Michal Vasina*[1,2]*, Nikola Velatova*[1]*, Lucia Cengelova*[1]*, David Kovar*[1,2]*, Jiri Damborsky*[1,2]*, Martin Marek*[1,2]*, David Bednar*[1,2]**, Zbynek Prokop*[1,2]***

[1]Loschmidt Laboratories, Department of Experimental Biology and RECETOX, Faculty of Science, Masaryk University, Brno, Czech Republic

[2]International Clinical Research Center, St. Anne's University Hospital, Brno, Czech Republic

Corresponding Author

*Contact information for the authors to whom correspondence should be addressed.

zbynek@chemi.muni.cz, 222755@mail.muni.cz

SUPPLEMENTARY FIGURES

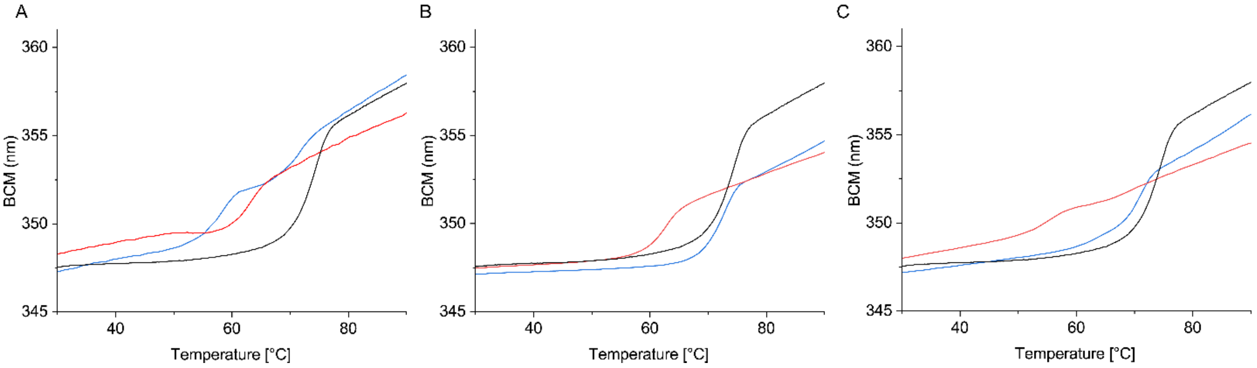

Supplementary Figure 1. Temperature denaturation of DhaA115 and mutant variants containing designed disulfide bridges. A) DhaA181, **B)** DhaA182, **C)** DhaA183. Each variant was measured in reducing (1 mM TCEP, red lines) and non-reducing (blue lines) conditions using DSF. Data for DhaA115 in non-reducing conditions are shown as black line. All experiments were recorded at 1°C/min scan rate.

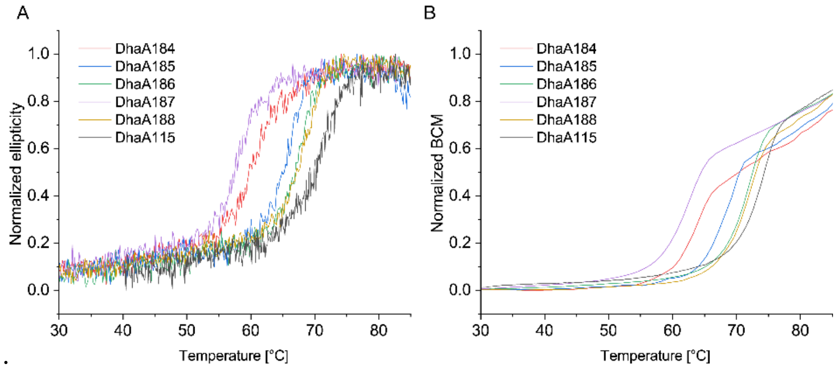

Supplementary Figure 2. Temperature denaturation of DhaA115 and its mutant variants designed by Rosetta. A) Normalized CD ellipticity at 227 nm and **B)** normalized barycentric mean of fluorescence spectra (BCM) during temperature scanning at 1 °C/min heating rate.

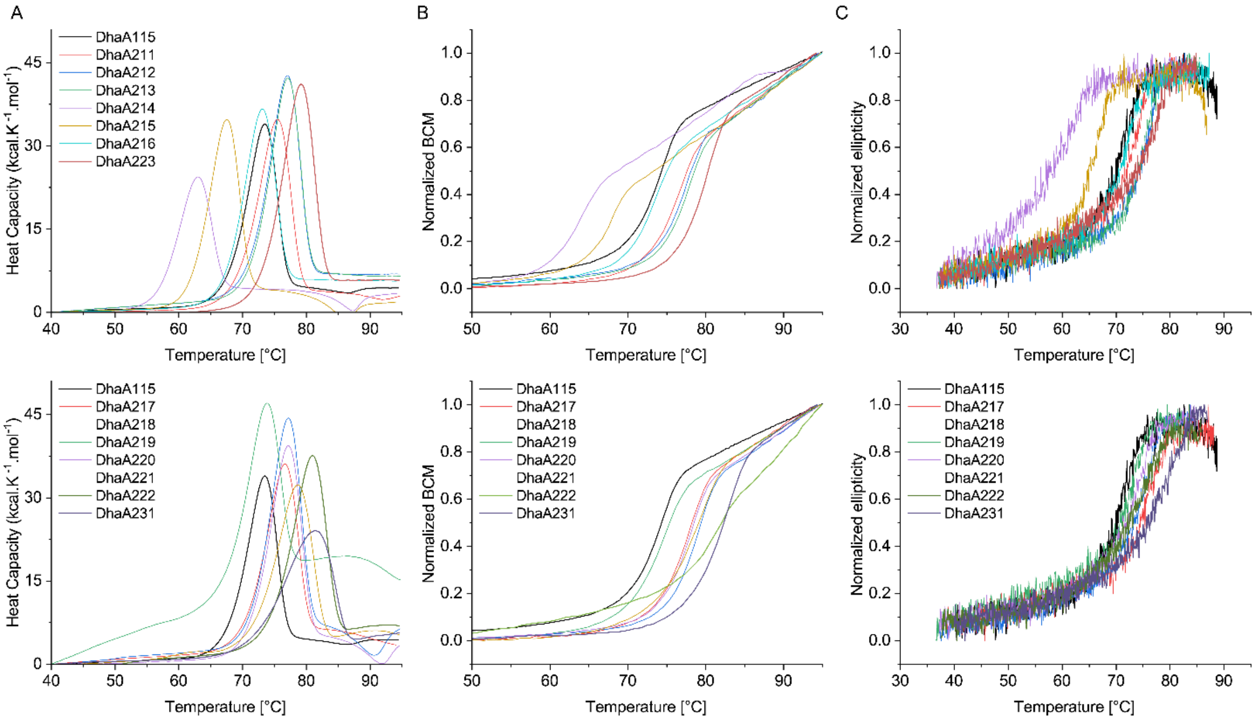

Supplementary Figure 3. Temperature denaturation of DhaA115 and its mutant variants designed by FireProt (top) and PROSS (bottom). A) DSC thermographs. **B)** Normalized BCM. **C)** Normalized CD ellipticity at 227 nm. All experiments were recorded at 1 °C/min scan rate.

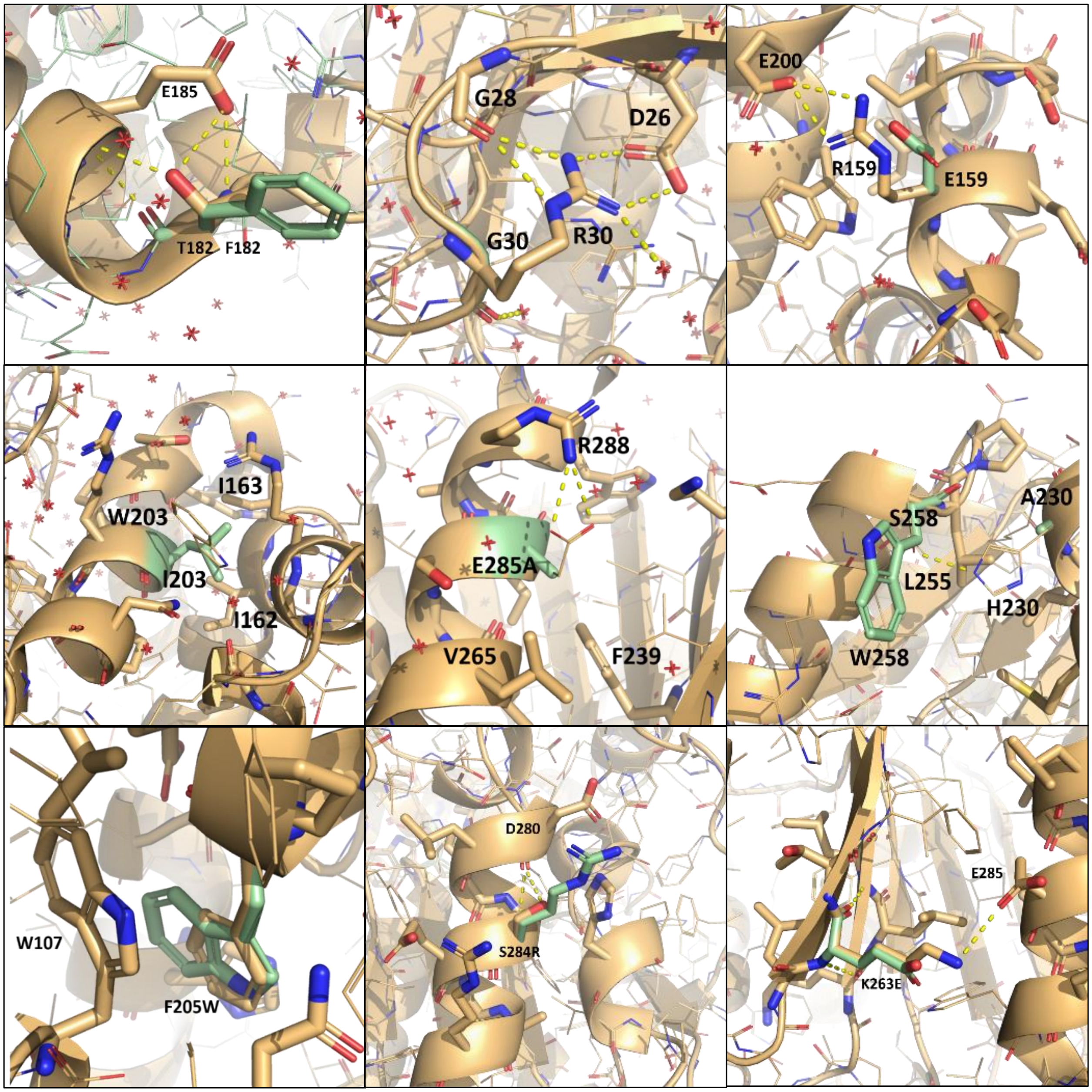

Supplementary Figure 4. Structural context of the potentially false positive predictions from FireProt and PROSS. Residues were mutated (green sticks) using the mutagenesis plugin of PyMOL and DhaA115 structure (PDB-ID: 6sp5).

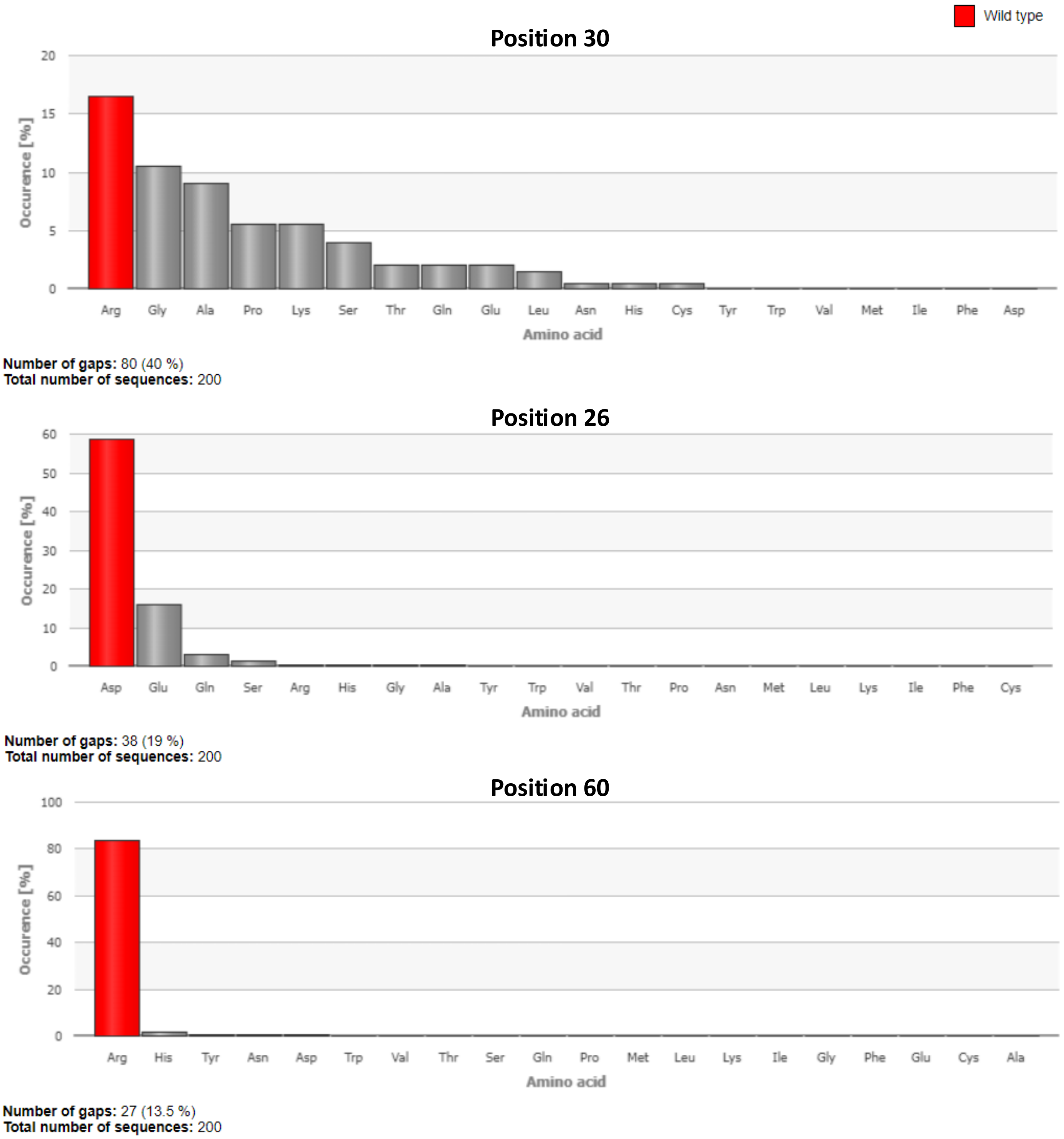

Supplementary Figure 5: Amino acid frequencies for positions 30 (top), 26 (center) and 60 (bottom) obtained by Hotspot Wizard for DhaA115 (PDB ID 6SP5), based on the multiple sequence alignment of 200 homologs.

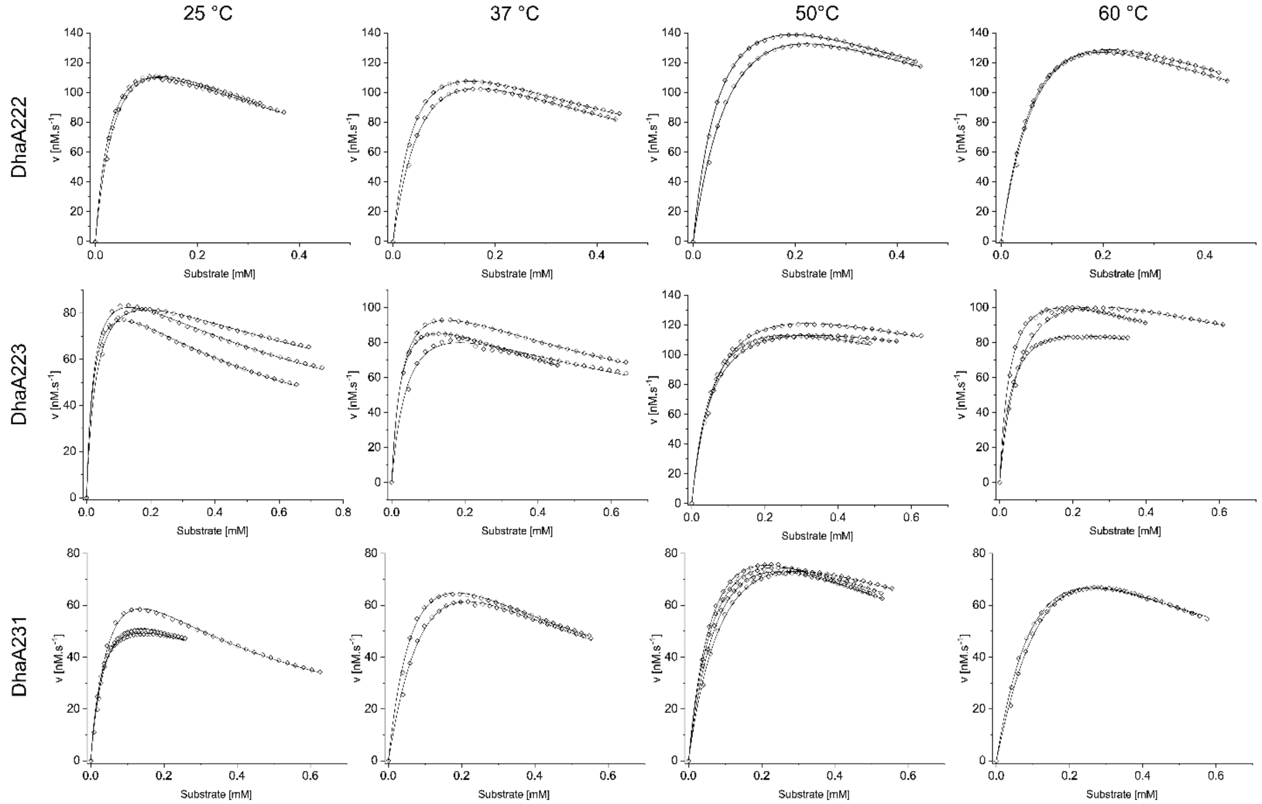

Supplementary Figure 6. Steady-state kinetics of DhaA222 (top), DhaA223 (middle), and DhaA231 (bottom) with 1,2-dibromoethane. The plots show reaction rates (diamonds) fitted to the Michaelis-Menten model with substrate inhibition (black lines) at different temperatures indicated on top of each panel. The enzyme concentrations were optimized for individual experiments to provide the best ITC signal and are different for each dataset.

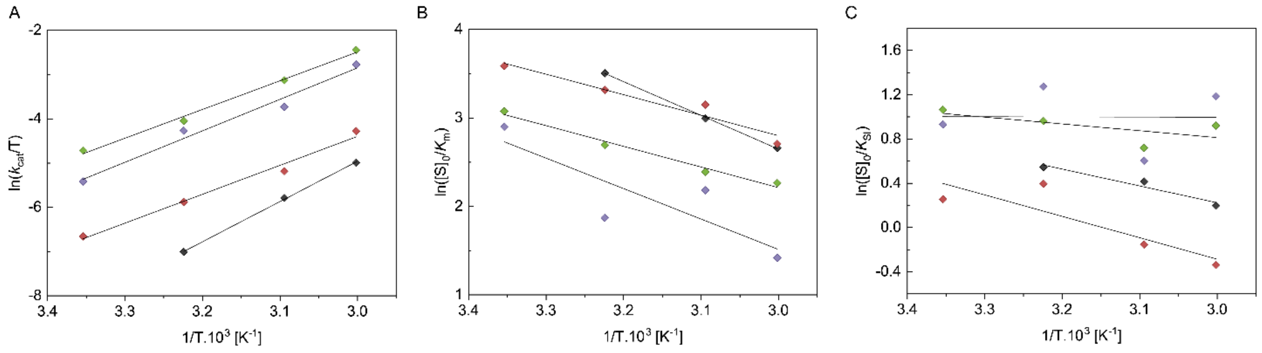

Supplementary Figure 7. Thermodynamic analysis of enzyme kinetics. A) Eyring plot of enzymatic turnover. **B)** van't Hoff's plots of K_m. **C)** van't Hoff's plots of K_{si} obtained from fits shown in **Supplementary Figure 5**. The parameters were fitted by linear fit (black line) to obtain the slopes and intercepts that were used to calculate thermodynamic parameters for DhaA222 (green), DhaA223 (red), DhaA231 (violet). Data for DhaA115 (black) are taken from our previous study [1].

SUPPLEMENTARY TABLES

Supplementary Table 1. List of residues selected for saturated mutagenesis using Rosetta.

Intra-domain		Inter-domain	
W138	M186	Y46	P201
R146	R190	G70	R204
Q150	F193	K74	E208
R153	R199	D78	P215
R159	E200	Y79	I218
I163	L202	F81	
N166	W203	H188	
I169	N207	E191	
E170		P192	
L173		W198	
P174		E200	

Supplementary Table 2. Results of the *in silico* saturation mutagenesis for the intra-domain region. Free energy stabilization (ΔΔG) predicted by Rosetta; mutability and top-3 frequency calculated by HotSpot Wizard. Only mutations with predicted stabilization (ΔΔG < 0) are shown. [a] colors according to the positions: red is closer to N-terminus, blue is closer to C-terminus; [b] color according to stabilization: darker green for the most negative energy, i.e. highest predicted stabilization effect; [c] stability ranging from 1 to 10, the higher meaning more mutable; [d] gap percentage in the alignment and the top-3 most frequent amino acids in each position.

Residue position[a]	Mutation	ΔΔG[b] (kcal/mol)	Ranking	Mutability[c]	Frequency[d]
153	R153W	-8.598	1	4	gap 75%, R 20%, G 1.5%, Q 1%,
207	N207H	-3.856	2	4	gap 83%, R 10.5%, N 3.5%, S 1.5%
153	R153M	-3.700	3		
207	N207Y	-3.275	4		
153	R153Q	-3.272	5		
153	R153F	-2.984	6		
153	R153I	-2.957	7		
207	N207F	-2.900	8		
153	R153T	-2.727	9		
207	N207W	-2.542	10		
153	R153V	-1.916	11		
153	R153L	-1.839	12		
153	R153S	-1.722	13		
207	N207V	-1.708	14		
153	R153N	-1.526	15		
186	M186F	-1.393	16	4	gap 77%, M 13%, R 4%, L 2.5%
146	R146L	-1.346	17	6	gap 59.5%, F 16%, R 11%, W 3.5%
153	R153H	-1.228	18		
207	N207E	-1.210	19		
153	R153G	-1.170	20		
146	R146I	-1.088	21		
153	R153C	-1.053	22		
150	Q150I	-0.976	23	6	gaps 60%, R 13.5%, N 12.5%, K 4%
146	R146V	-0.967	24		
153	R153A	-0.959	25		
169	I169L	-0.951	26	5	gap 72%, V13.5%, I 10.5%, L 1.5%

207	N207T	-0.940	27		
207	N207I	-0.895	28		
146	R146F	-0.861	29		
207	N207A	-0.816	30		
207	N207Q	-0.813	31		
169	I169V	-0.730	32		
166	N166L	-0.699	33	4	gap 73%, N 22%, R 1%, L 0.5%
159	R159W	-0.670	34	3	gaps 98%, R 1%, K 0.5%, E 0.5%
207	N207C	-0.580	35		
153	R153Y	-0.535	36		
173	L173L	-0.534	37	6	gaps 74%, L 16.5%, V 4.5%, M 1%
166	N166N	-0.528	38		
146	R146R	-0.515	39		
193	F193W	-0.509	40	2	gaps 78%, F 15.5%, Y 6%
159	R159F	-0.503	41		
186	M186L	-0.488	42		
207	N207S	-0.456	43		
186	M186M	-0.389	44		
153	R153R	-0.352	45		
193	F193F	-0.314	46		
207	N207L	-0.226	47		
150	Q150M	-0.220	48		
203	W203F	-0.168	49	4	gaps 84%, L 11.5%, W 3.5%, F 0.5%
146	R146A	-0.167	50		
190	R190R	-0.156	51	5	gaps 78%, R 15.5%, L 2%, D 2%
146	R146Q	-0.105	52		
202	L202M	-0.099	53	5	gaps 84%, T 7.5%, L 3.5%, M 2.5%
166	N166G	-0.095	54		
163	I163I	-0.049	55	6	gaps 69%, L14%, I 4.5%, A2.5%
150	Q150Q	-0.032	56		
207	N207N	-0.027	57		

Supplementary Table 3. Results of the *in silico* saturation mutagenesis for the inter-domain region. Free energy stabilization (ΔΔG) predicted by Rosetta; mutability and top-3 frequency calculated by HotSpot Wizard. Only mutations with predicted stabilization (ΔΔG < 0) are shown. [a] colors according to the positions: red is closer to N-terminus, blue is closer to C-terminus; [b] color according to stabilization: darker green for the most negative energy, i.e. highest predicted stabilization effect; [c] stability ranging from 1 to 10, the higher meaning more mutable; [d] gap percentage in the alignment and the top-3 most frequent amino acids in each position.

Residue position[a]	Mutation	ΔΔG[b] (kcal/mol)	Ranking	Mutability[c]	Frequency[d]
74	K74F	-9.416	1	4	gaps 15.5%, K 66.5%, A %, R 4.5%
74	K74Y	-9.315	2		
74	K74W	-7.985	3		
74	K74I	-5.191	4		
74	K74M	-5.036	5		
74	K74H	-4.894	6		
208	E208Y	-4.441	7	3	gaps 83%, E 10.5%, Q 5%, S 0.5%
74	K74V	-4.388	8		
208	E208L	-4.279	9		
74	K74Q	-4.174	10		
204	R204W	-4.128	11	6	gaps 84%, T 9.5%, V 2%, R 1.5%, W 0.5%
208	E208M	-4.117	12		
74	K74S	-3.591	13		
208	E208H	-3.343	14		
74	K74N	-3.231	15		
191	E191Y	-2.906	16	5	gaps 78.5%, R 8.5%, A 7%, E 3.5%, Y 0.5%
204	R204M	-2.751	17		
204	R204F	-2.698	18		
208	E208F	-2.406	19		
74	K74L	-2.293	20		
208	E208W	-2.271	21		
188	H188W	-2.248	22	7	gaps 77.5%, E 6%, V 4.5%, A 4%
188	H188Y	-2.244	23		
204	R204Y	-2.239	24		
74	K74A	-2.223	25		
191	E191F	-2.168	26		

191	E191W	-2.109	27		
74	K74C	-2.069	28		
78	D78T	-1.998	29	7	gaps 19%, E 30.5%, S 9%, A 8%, T 3%
208	E208N	-1.958	30		
74	K74G	-1.581	31		
208	E208Q	-1.577	32		
78	D78S	-1.562	33		
188	H188H	-1.473	34		
204	R204N	-1.470	35		
191	E191N	-1.376	36		
70	G70G	-1.327	37	2	gaps 15%, G 82.5%, N 2%, S 0.5%
204	R204Q	-1.327	38		
74	K74R	-1.278	39		
74	K74T	-1.179	40		
188	H188Q	-1.067	41		
215	P215P	-1.052	42	2	gaps 85%, P 14.5%, T 0.5%
46	Y46F	-1.022	43	4	gaps 16%, Y 58%, F 17%, H 3%
191	E191I	-1.006	44		
74	K74D	-0.974	45		
81	F81F	-0.941	46		
74	K74K	-0.895	47		
46	Y46Y	-0.893	48		
78	D78D	-0.868	49		
188	H188F	-0.840	50		
78	D78H	-0.806	51		
191	E191M	-0.770	52		
208	E208E	-0.668	53		
204	R204V	-0.654	54		
218	I218I	-0.614	55	5	gaps 85%, V 7.5, T 2.5%, M 2%, F 0.5%
191	E191Q	-0.485	56		
78	D78A	-0.443	57		
191	E191E	-0.342	58		
191	E191V	-0.327	59		
78	D78E	-0.290	60		
191	E191A	-0.258	61		
188	H188M	-0.246	62		
191	E191R	-0.237	63		
208	E208A	-0.206	64		
218	I218F	-0.176	65		
192	P192P	-0.148	66	3	gaps 78%, P 20%, T 0.5%, L 0.5%
200	E200Y	-0.147	67	4	gaps 83%, R 9.5%, K 3%, E 2.5%

78	D78L	-0.059	68		
79	Y79F	-0.043	69	3	gaps 18.5%, Y65%, H 10.5 %, F 2%

Supplementary Table 4: List of DhaA115 mutations proposed by FireProt webserver.

		**Energy mutant:** -5.33 kcal/mol (3 mutations)			
**Position**	**Mutation**	**Conserved**	**Correlated**	**ΔΔG FoldX[b]**	**ΔΔG Rosetta[b]**
212	A212N	N	N	-1.16	-3.63
165	Q165Y	N	N	-1.30	-3.37
171	G171I	N	N	-1.43	-2.90
		**Evolution mutant:** 1.65 kcal/mol (10 mutations)			
**Position**	**Mutation[a]**	**BTC by ratio**	**BTC by majority**	**ΔΔG FoldX[b]**	
30	R30G	Y	Y	0.14	
184	V184E	Y	Y	-0.63	
197	V197E	Y	N	-0.36	
217	N217D	Y	Y	-0.85	
119	N119H	N	Y	-1.13	
159	R159E	N	Y	-0.48	
161	L161M	N	Y	0.45	
163	I163L	N	Y	-0.07	
203	W203L	N	Y	0.00	
285	E285A	N	Y	-0.12	

[a]Orange mutations are predicted by energy approach, green ones are predicted from back to consensus by ratio, blue ones are predicted from back to consensus by majority; [b]in kcal/mol; negative ΔΔG values are stabilizing mutations; positive ΔΔG are destabilizing.

Supplementary Table 5: List of DhaA115 designs suggested by PROSS webserver.

Position	DhaA115	*PROSS designs*								
		1	***2***[a]	*3*	*4*	***5***[a]	*6*	*7*	***8***[a]	*9*
197	V	E	**E**	E	E	**E**	E	E	**E**	E
184	V	E	**E**	E	E	**E**	E	E	**E**	E
258	S	W	**W**	W	W	**W**	W	W	**W**	W
217	N	D	**D**	D	D	**D**	D	D	**D**	D
119	N	H	**H**	H	H	**H**	H	H	**H**	H
242	T		**E**	E	E	**E**	E	E	**E**	E
30	R		**P**	P	P	**P**	P	P	**P**	P
227	N		**R**	R	R	**R**	R	R	**R**	R
124	K		**R**	R	R	**R**	R	R	**R**	R
230	H		**A**	A	A	**A**	A	A	**A**	A
11	D		**P**	P	P	**P**	P	P	**P**	P
254	R			W	W	**W**	W	W	**W**	W
212	A			D	D	**D**	G	G	**G**	G
218	I				V	**V**	V	V	**V**	V
205	F			W	W	**W**	W	W	**W**	
284	S					**R**	R	R	**R**	R
221	L					**I**	I	I	**I**	I
263	K					**E**	E	E	**E**	E
156	D					**G**	G	G	**G**	G
281	L						E	E	**E**	E
255	L						C	C	**C**	C
7	G						D	S	**S**	D
167	A						M	M	**M**	M
175	K							A	**A**	A
117	K							R	**R**	M
164	D							E	**E**	E
58	S								**T**	T
150	Q								**K**	R
161	L								**M**	M
171	G								**Q**	Q
99	E								**D**	N
160	E								**K**	K
82	D								**E**	E
256	A								**R**	R
253	A								**E**	E
250	A								**E**	E
178	V									M
264	T									V
231	Q									T
233	P									D
71	K									R
135	I									M
144	F									A
251	E									Q
191	E									A
121	E									D
280	D									H
151	A									K

[a]Designs suggested for characterization.

Supplementary Table 6: Frequencies (%) of mutations that were removed from FireProt or PROSS variants based on manual curation. The frequencies were calculated from MSA of 200 sequences by HotSpot Wizard using DhaA115 as input. Original (bold black) and mutated (bold red) amino acids are highlighted in boxes.

	R30G/P	N119H	W203L	E285A	H230A	S258W	F205W	R254W	K263E	S284E
**A**	9.0	2.5	1.0	**6.0**	**17.0**	2.0	0.0	0.0	0.5	4.0
**C**	0.5	1.0	0.0	0.0	1.0	0.0	0.0	0.0	0.0	0.0
**D**	0.0	1.0	0.0	0.0	0.0	1.0	0.0	2.5	3.5	1.0
**E**	2.0	0.0	0.0	**3.5**	2.5	0.5	0.0	4.5	**11.0**	**4.5**
**F**	0.0	0.5	1.0	0.0	0.5	0.0	**22.5**	0.0	0.0	0.0
**G**	**10.5**	0.0	0.0	3.0	1.5	2.0	0.5	1.0	0.0	0.0
**H**	0.5	**36.5**	1.5	1.0	**0.5**	6.5	0.0	0.0	0.5	0.5
**I**	0.0	0.5	0.0	0.0	0.5	0.5	0.5	0.5	0.0	0.0
**K**	5.5	0.5	0.0	0.5	2.0	1.5	0.0	2.0	**4.5**	0.5
**L**	1.5	0.5	32.0	0.0	3.5	0.5	1.0	0.0	0.0	0.0
**L**	1.5	0.5	**32.0**	0.0	3.5	0.5	1.0	0.0	0.0	0.0
**M**	0.0	8.5	0.0	0.0	0.0	0.0	2.0	0.0	0.0	0.0
**N**	0.5	**15.5**	0.0	0.5	0.0	7.5	0.0	0.5	0.0	0.0
**P**	**5.5**	0.0	0.0	0.0	0.5	0.0	0.0	0.5	0.0	0.0
**Q**	2.0	6.5	0.0	0.0	0.5	0.0	0.0	1.0	0.5	2.0
**R**	**16.5**	7.5	14.5	1.0	1.0	1.0	0.0	**1.0**	4.5	1.5
**S**	4.0	0.5	0.0	0.5	4.5	**3.5**	0.0	1.0	1.0	**1.5**
**V**	0.0	0.5	0.0	0.0	0.0	0.0	0.0	0.0	0.5	0.5
**W**	0.0	0.0	**5.0**	0.0	0.0	**0.0**	**26.5**	**14.5**	0.0	0.0
**Y**	0.0	2.0	1.5	0.5	0.0	0.0	0.0	1.0	0.0	0.0
**-**	39.5	15.0	43.0	81.5	63.5	69.5	41.0	69.5	69.5	81.5

Supplementary Table 7: Correlated positions found in DhaA115 by HotSpot Wizzard. The threshold for consensus score which concatenates evaluation from several different tools were set to default (3.5).

Position 1	Residue 1	Mutability	Position 2	Residue 2	Mutability	Consensus score
41	N	4	48	W	1	3.95
44	S	3	51	I	5	4.48
			87	Y	5	3.90
			110	A	6	3.53
48	W	1	51	I	5	3.96
51	I	5	110	A	6	3.80
			105	H	2	3.63
81	F	5	87	Y	5	3.92
87	Y	5	114	H	6	3.52
109	S	4	131	F	5	3.96
110	A	6	114	H	6	3.73
170	E	5	190	R	5	3.70

Supplementary Table 8: MutCompute analysis of all mutations experimentally analyzed within this study. The score is given as a logarithm of ratios between probabilities of finding the original residue (P_{wt}) and the newly introduced mutation (P_{mut}) in the given structural context. Stabilizing mutations are positive (green), destabilizing are negative (red). The most likely residue to be found in the given position based on MutCompute is also shown (P_{best}).

Position	Original residue	Designed mutation	Software	DhaA variant	MutCompute predictions		
					Log(P_{mut}/P_{wt})	Best residue	Log(P_{best}/P_{wt})
7	G	S	PROSS	221, 222, 231	-2.8	G	0.0
11	D	P	PROSS	217-222	-3.4	A	0.7
**30**	**R**	**G**	**FP**	**212**	**-7.1**	R	0.0
		**P**	**PROSS**	**217, 219, 221**	**-6.6**		
58	S	T	PROSS	221,222	-2.1	S	0.0
74	K	F	FP	184	-3.5	R	0.0
79	Y	C	DbD2	181	-7.2	F	0.4
82	D	E	PROSS	221,222,231	-1.1	A	0.7
99	E	D	PROSS	221,222,231	0.9	D	0.9
117	K	R	PROSS	221,222,231	-0.8	M	2.3
**119**	**N**	**H**	**PROSS**	**217,219,221**	**-3.0**	N	0.0
124	K	R	PROSS	217-222,231	-0.5	K	0.0
150	Q	I	FP	186-189	-6.7	Q	0.0
		K	PROSS	221,222,231	-1.1		
156	D	G	PROSS	219-222,231	-1.5	N	0.1
160	E	K	PROSS	221,222,231	0.5	K	0.5
161	L	M	FP	214-216	-4.8	L	0.0
			PROSS	221,222,231			
162	I	C	DbD2	182,183	-4.8	I	0.0
163	I	L	FP	214-216	-4.4	I	0.0
164	D	E	PROSS	221,222,231	0.1	E	0.1
165	Q	Y	FP	211,216,223	-2.9	Q	0.0
167	A	M	PROSS	221,222,231	-5.6	A	0.0
169	I	L	Rosetta	186,187,189	-1.8	V	0.4
171	G	I	FP	211,216,223	-6.5	G	0.0
		Q	PROSS	221,222,231	-3.8		
175	K	A	PROSS	221,222,231	-3.4	M	1.3
184	V	E	FP	212,213,216,223	-1.4	T	0.1
			PROSS	217-222,231			
186	M	F	Rosetta	187,189	-5.0	M	0.0
188	H	W	Rosetta	188,189	-2.9	H	0.0
197	V	E	FP	212,213,216,223	1.3	S	2.5
			PROSS	217-222,231			
201	P	C	DbD2	181,183	-8.4	P	0.0
**203**	**W**	**C**	**DbD2**	**182,183**	**-9.9**	W	0.0
		**L**	**FP**	**214**	**-8.9**		
204	R	W	Rosetta	188,189	-3.3	R	0.0
**205**	**F**	**W**	**PROSS**	**219,221**	**-0.7**	**F**	0.0

207	N	H	Rosetta	187,189	-4.0	N	0.0
208	E	Y	Rosetta	185	-3.9	E	0.0
212	A	N	FP	211,216,223	-4.0	A	0.0
		D	PROSS	219-222	-3.1		
217	N	D	FP	212,213,216,223	0.8	D	0.8
			PROSS	217-222,231			
218	I	V	PROSS	219-222,231	-2.2	I	0.0
221	L	I	PROSS	219-222	-4.8	L	0.0
227	N	R	PROSS	217-222	-0.6	K	0.9
**230**	**H**	**A**	**PROSS**	**217,219,221**	**-3.7**	K	0.6
242	T	E	PROSS	217-222,231	-0.3	T	0.0
250	A	E	PROSS	221,222,231	-0.3	A	0.0
253	A	E	PROSS	221,222,231	-1.3	A	0.0
**254**	**R**	**W**	**PROSS**	**219,221**	**-6.3**	R	0.0
255	L	C	PROSS	221,222,231	-3.5	L	0.0
256	A	R	PROSS	221,222,231	-5.6	A	0.0
**258**	**S**	**W**	**PROSS**	**217,219,221**	**-7.5**	S	0.0
**263**	**K**	**E**	**PROSS**	**219,221**	**-3.6**	K	0.0
281	L	E	PROSS	221	-1.7	L	0.0
**284**	**S**	**R**	**PROSS**	**219,221**	**-4.2**	S	0.0
**285**	**E**	**A**	**FP**	**214**	**-6.3**	E	0.0

Supplementary Table 9. Temperature-induced denaturation of DhaA115, 222, 223, and 231. The apparent melting temperatures (T_m^{app}) were estimated from inflection points (CD, DSF) or peak maxima (DSC) of the unfolding curves. Values are means and standard deviations of three independent measurements (n=3). The values of calorimetric enthalpy (ΔH_{cal}) and energy barrier of the first unfolding step ($\Delta G^{\ddagger}$) were obtained by global fitting of multiple experimental datasets to the biologically relevant two-step irreversible unfolding model.

Protein	T_m^{app} [°C]			ΔH_{cal} [kJ/mol]	$\Delta G^{\ddagger}_1$ [kJ.mol^{-1}]	
	**DSC**	**CD**	**DSF**		**(70°C)**	**(80°C)**
DhaA115	73.5 ± 0.0	71.3 ± 0.0	75.0 ± 0.0	746 ± 28	103 ± 0	93 ± 0
DhaA222	80.9 ± 0.0	80.3 ± 1.4	81.6 ± 0.1	889 ± 57	108 ± 0	101 ± 0
DhaA223	79.1 ± 0.0	78.5 ± 1.0	80.3 ± 0.0	953 ± 36	110 ± 0	101 ± 0
DhaA231	81.3 ± 0.0	81.0 ± 0.0	83.0 ± 0.0	793 ± 32	108 ± 0	101 ± 0

Supplementary Table 10. Urea-induced denaturation of DhaA115, 222, 223, and 231. In the chemical denaturation experiments, the first three SVD components were fitted globally to the two-step reversible model. C_m – urea midpoint of the transition, m – m-value defining the steepness of the transition, ΔG – Gibbs free energy of unfolding at 25 °C.

Protein	C_{m1} [M]	m_1 [kJ.mol^{-1}.M^{-1}]	C_{m2} [M]	m_2 [kJ.mol^{-1}.M^{-1}]	ΔG_{NI} [kJ.mol^{-1}]	ΔG_{ID} [kJ.mol^{-1}]
DhaA115	3.7 ± 0.0	9.8 ± 0.9	5.4 ± 0.0	5.6 ± 0.2	36.6 ± 3.3	30.1 ± 1.0
DhaA222	4.2 ± 0.1	7.4 ± 1.5	5.7 ± 0.1	8.2 ± 1.2	31.4 ± 5.5	47.0 ± 7.0
DhaA223	5.2 ± 0.1	9.1 ± 2.2	6.3 ± 0.0	11.9 ± 1.9	47.2 ± 11.0	74.8 ± 12.0
DhaA231	5.6 ± 0.3	8.2 ± 2.7	6.7 ± 0.1	6.4 ± 2.5	46.0 ± 13.8	43.0 ± 16.8
DhaA231*	5.8 ± 0.1	9.6 ± 2.0			55.7 ± 11.5	

Supplementary Table 11. Crystallographic data collection and refinement statistics.

Data collection*	DhaA223	DhaA231
Wavelength (Å)	0.999	0.999
Space group	$P12_11$	$P2_12_12_1$
Cell dimensions		
a, b, c (Å)	67.35, 143.14, 106.68	44.43 67.99 122.38
α, β, γ (°)	90, 108.39, 90	90, 90, 90
Resolution (Å)	47.72 – 1.51 (1.56 – 1.51)	45.48 – 1.31 (1.35 – 1.31)
Total reflections	1030201 (97885)	1051356 (46983)
Unique reflections	296139 (29119)	90140 (8332)
Rmerge	9.9 (105.3)	5.2 (118)
I / σI	7.75 (1.2)	20.5 (1)
Completeness (%)	98.1 (96.7)	99.1 (92.8)
Multiplicity	3.5 (3.4)	11.7 (5.6)
CC(1/2)	99.7 (54.2)	100 (55.7)
Wilson B-factor	13.9	18.7
**Refinement**		
Resolution (Å)	47.72 – 1.51 (1.56 – 1.51)	45.48 – 1.31 (1.35 – 1.31)
No. reflections	296124 (29091)	90139 (8323)
Rwork / Rfree (%)	16.8 / 19.5	15.4 / 16.8
Number of atoms		
Protein	14457	2432
Ligand	160	5
Water	1855	420
B-factors		
Protein	17.2	24.0
Ligand	36.4	27.6
Water	30.4	37.9
R.m.s deviations		
Bond lengths (Å)	0.017	0.015
Bond angles (º)	1.44	1.37
Ramachandran favored (%)	96.54	96.89
Ramachandran allowed (%)	3.46	3.11
Ramachandran outliers (%)	0	0
PDB ID code	8OE2	8OE6

*Values in parentheses are for the highest-resolution shell.

Supplementary Table 12. Table of root-mean-square deviation (RMSD) values between structures of DhaA115, DhaA223 and DhaA231.

	RMSD values		
DhaA115	0.0	0.4	0.7
DhaA223	0.4	0.0	0.7
DhaA231	0.7	0.7	0.0

Supplementary Table 13. Specific activities of DhaA115 and its stabilized variants towards 1,2-dibromoethane measured by Iwasaki assay. The provided values are means of three independent measurements with standard deviations.

Protein	Specific activity [nmol.s^{-1}.mg^{-1}]	
	37°C	70°C
DhaA115	7.6 ± 0.5	17.1 ± 2.5
DhaA222	18.5 ± 0.0	22.0 ± 4.9
DhaA223	7.6 ± 0.9	38.5 ± 7.7
DhaA231	13.6 ± 2.0	23.9 ± 2.5

Supplementary Table 14. Panel of standard 27 substrates used for characterization of haloalkane dehalogenase substrate specificity. The substrates whose dehalogenation could not be assayed due to incompatibility with the MicroPEX platform are marked in red.

#	Substrate name	#	Substrate name
S4	1-chlorobutane	S67	1,2-dichloropropane
S6	1-chlorohexane	S72	1,2-dibromopropane
S18	1-bromobutane	S80	1,2,3-trichloropropane
S20	1-bromohexane	S111	bis(2-chloroethyl)ether
S28	1-iodopropane	S115	1-chlorocyclohexane
S29	1-iodobutane	S117	1-bromocyclohexane
S31	1-iodohexane	S119	1-(bromomethyl)cyclohexane
S37	1,2-dichloroethane	S137	1-bromo-2-chloroethane
S38	1,3-dichloropropane	S138	1-chlorocyclopentane
S40	1,5-dichloropentane	S141	4-bromobutyronitrile
S47	1,2-dibromoethane	S154	1,2,3-tribromopropane
S48	1,3-dibromopropane	S155	1,2-dibromo-3-chloropropane
S52	1-bromo-3-chloropropane	S209	3-chloro-2-methylpropane
S54	1,3-diiodopropane		

Supplementary Table 15. Kinetic constants from fitting of steady-state conversion of 1,2-dibromoethane by the selected DhaA variants. K_m – Michaelis-Menten constant, K_{si} – substrate inhibition constant, k_{cat} – enzyme turnover. The provided values are means of two or three independent measurements with standard deviations. Data for DhaA115 are taken from Markova et al., 2021.

DhaA	T [°C]	k_{cat} [s^{-1}]	K_m [mM]	K_{si} [mM]	k_{cat}/K_m [$s^{-1}mM^{-1}$]	$k_{cat}K_{si}/K_m$ [s^{-1}]
115	37	0.280 ± 0.002	0.030 ± 0.001	0.58 ± 0.02	9.3 ± 0.3	5.4 ± 0.3
	50	0.988 ± 0.027	0.050 ± 0.002	0.66 ± 0.01	19.8 ± 1.0	13.0 ± 0.7
	60	2.266 ± 0.038	0.070 ± 0.003	0.82 ± 0.04	32.4 ± 1.5	26.5 ± 1.8
222	25	2.670 ± 0.219	0.046 ± 0.011	0.34 ± 0.04	57.9 ± 15.1	19.9 ± 5.7
	37	5.420 ± 0.491	0.068 ± 0.023	0.38 ± 0.08	80.0 ± 27.7	30.6 ± 12.3
	50	14.168 ± 1.264	0.092 ± 0.032	0.49 ± 0.08	154.5 ± 55.7	75.1 ± 29.9
	60	28.910 ± 0.568	0.104 ± 0.007	0.40 ± 0.01	277.8 ± 19.8	110.6 ± 8.5
223	25	0.384 ± 0.003	0.028 ± 0.005	0.77 ± 0.26	13.9 ± 2.6	10.7 ± 4.1
	37	0.867 ± 0.035	0.036 ± 0.012	0.67 ± 0.07	23.9 ± 8.1	16.1 ± 5.7
	50	1.815 ± 0.261	0.043 ± 0.017	1.17 ± 0.19	42.3 ± 18.1	49.3 ± 22.6
	60	4.625 ± 0.355	0.067 ± 0.009	1.40 ± 0.35	69.3 ± 10.5	97.1 ± 28.4
231	25	1.319 ± 0.349	0.055 ± 0.020	0.39 ± 0.17	24.0 ± 11.0	9.5 ± 6.0
	37	4.337 ± 0.792	0.154 ± 0.071	0.28 ± 0.07	28.2 ± 14.0	7.9 ± 4.4
	50	7.776 ± 0.329	0.113 ± 0.024	0.55 ± 0.07	68.9 ± 14.7	37.6 ± 9.5
	60	20.852 ± 4.833	0.242 ± 0.094	0.31 ± 0.08	86.1 ± 38.8	26.3 ± 13.9

REFERENCES

1. Markova, K.; Kunka, A.; Chmelova, K.; Havlasek, M.; Babkova, P.; Marques, S. M.; Vasina, M.; Planas-Iglesias, J.; Chaloupkova, R.; Bednar, D.; Prokop, Z.; Damborsky, J.; Marek, M. Computational Enzyme Stabilization Can Affect Folding Energy Landscapes and Lead to Catalytically Enhanced Domain-Swapped Dimers. ACS Catalysis. 2021 5;11(21):12864–85.
