# Supplementary material for: Advancing Enzyme’s Stability and Catalytic Efficiency through Synergy of Force-Field Calculations, Evolutionary Analysis, and Machine Learning
Source: ACS Catal. 2023 Sep 11;13(19):12506–18. doi: 10.1021/acscatal.3c02575 (PMC10563018; doi:10.1021/acscatal.3c02575)
Supplement: Supplementary file 7 — cs3c02575_si_007.pdf [file cs3c02575_si_007.pdf]

SUPPLEMENTARY MATERIALS AND METHODS

Advancing Enzyme´s Stability and Catalytic Efficiency through Synergy of Force-Field Calculations, Evolutionary Analysis and Machine Learning

Antonin Kunka[1,2]‡†, Sergio M. Marques[1,2]‡, Martin Havlasek[1], Michal Vasina[1,2], Nikola Velatova[1], Lucia Cengelova[1], David Kovar[1,2], Jiri Damborsky[1,2], Martin Marek[1,2], David Bednar[1,2], Zbynek Prokop[1,2]**

[1]Loschmidt Laboratories, Department of Experimental Biology and RECETOX, Faculty of Science, Masaryk University, Brno, Czech Republic

[2]International Clinical Research Center, St. Anne's University Hospital, Brno, Czech Republic

Corresponding Author

*Contact information for the authors to whom correspondence should be addressed.

zbynek@chemi.muni.cz, 222755@mail.muni.cz

COMPUTATIONAL DESIGN

Rational design of disulfide bridges

The Disulfide by Design 2.0 [1] webserver was used to predict potential favorable cysteine mutations to introduce into DhaA115. Towards this, protonated crystal structure of DhaA115 (PDB ID: 6SP5, [2]) was uploaded to the webserver and the calculations were run using the default settings.

Saturation mutagenesis by Rosetta

Two regions were selected for mutagenesis based on the visual evaluation of the DhaA115 (PDB ID: 6SP5) crystal structure: (i) intra-domain interface of the cap domain, and (ii) inter-domain interface between the cap domain and the hydrolase core (Table S1). Within these interface regions, the residues interacting with different secondary elements were selected for saturated *in silico* mutagenesis. The water molecules and ions were removed from the crystal structure and the protein chain was renumbered to start from position 1. The resulting structure was minimized by Rosetta using *minimize_with_cst* module. The minimization was performed according to ref. [3]. Both backbone and side chain optimization were enabled (*sc_min_only false*), the distance for full atom pair potential was set to 9 Å (*fa_max_dis 9.0*), the standard weights for the individual terms in the energy function were used with constraint weight 1 (*constraint_weight* 1.0). The output from the minimization step was used by the script *convert_to_cst_file.sh* for creation of the constraints file. To calculate the most stable conformers of each mutant, Protocol 16 was followed. For that, *ddg_monomer* module of Rosetta was used according to ref. [3], incorporating the backbone flexibility. The soft-repulsive design energy function (*soft_rep_design weights*) was used for side chains repacking and backbone minimization (*sc_min_only false*). The optimization was performed on the whole protein without distance restriction (*local_opt_only false*). The previously generated constraints *cst* file was used during minimization (*min_cst true*) to impose a restraint of

0.5 Å on the C_α atoms. The optimization was performed in three rounds with increasing weight on the repulsive term (*ramp_repulsive* true). The structure with the lowest energy (*mean false*, *min true*) was selected from the 50-iteration cycle (*iterations 50*), and it was used as the final result to obtain the minimized model of the mutant. All calculations used *talaris2014* [4,5] force field. All targeted positions were mutated to all 20 amino acids. To verify the mutability and conservation of the selected positions, the HotSpot Wizard 3.0 webserver was used [6,7] with default settings. The top-ranked mutations based on Rosetta were visually inspected and evaluated according to the respective mutability and conservation analysis, as assessed by HotSpot Wizard. The riskiest mutations based on this analysis (i.e., likely to be false positives) were experimentally characterized as single-point mutants. while others were considered safer (i.e., less likely to be false positives) were combined.

Mutation design by FireProt and PROSS webservers

FireProt [8] and PROSS [9] web-tools were used to find stabilizing multiple-point mutations on DhaA115 employing both evolutionary and energy-based approaches. Both methods construct a multiple sequence alignment to find non-conserved positions suitable for mutagenesis, and both use Rosetta to predict the free energy stabilization (ΔΔG) introduced by the different substitutions. FireProt also runs energy calculations with FoldX calculations prior to Rosetta, or just FoldX in the case of the evolution-based mutations). The DhaA115 crystal structure was specified by the respective PDB ID (6SP5, chain A) in both tools.

FireProt used the default setting: BLAST E-value 1×10^{-10}, maximum number of sequences of 200, minimal identity of 30% and maximal identity of 90%. The catalytic residues N41, D106, W107, E130 and H272 were excluded. The webserver proposed one energy-based, and two evolutionary-based multiple-point mutant variants which were selected for experimental

characterization. Moreover, all suggested mutations were further evaluated as described in the sections below, and those that passed were combined.

PROSS used the default settings: minimal sequence identity of 35%, minimal homologue sequence coverage of 75%, maximum number of initial target search of 4000, BLASTp E-value 1×10^{-4}, Rosetta force field *Talaris2014*. The webserver provided a total of 9 designs from which 3 were selected following the authors' guidelines, i.e., we started with a design that comprises ca. 10 % mutations (design 8) and then went down to a design that comprises around 10 mutations. Similarly to FireProt, all mutations were evaluated as described below and those that did not pass were removed from the suggested designs to yield additional "safe" variants for experimental characterization.

Manual curation of the mutations

The mutations suggested by FireProt and PROSS were manually curated by their visual inspection in PyMOL 2.3.2 [10] and the Missense3D server [11] to assess any potential damage introduced to the structures. We used TANGO [12] and Aggrescan3D 2.0 [13] tools to predict the aggregation propensity, carried out using the DhaA115 structure (PDB ID: 6SP5) with the dynamic mode ON and the distance of aggregation analysis set to 5 Å.

Mutation evaluation by MutCompute and HotSpot Wizard

Mutations in DhaA222 designed by PROSS were further evaluated by convolutional neural network MutCompute [14] and HotSpotWizard (HSW, [6,7]). Crystal structure of DhaA115 (PDB ID: 6SP5) was analyzed using the webservers with default settings. Mutations that had low probability of substitution based on the MutCompute, and low mutability score according to HSW were eliminated from DhaA222.

Molecular dynamics simulations and analysis

The crystal structures of DhaA115 (PDB ID: 6SP5), DhaA223 and DhaA231 (this work) were used as starting points. The solvent, co-crystallization molecules, and ions were removed, and the double side chains were corrected to keep only the most populated conformations using the *pdb4amber* module of AmberTools 16 [15]. The hydrogen atoms were predicted using the H++ server [16] calculated in an implicit solvent at both pH 7.5 and 10.5, 0.1 M salinity, an internal dielectric constant of 10, and external of 80. The original crystallization solvent was added and the *tLEaP* program of AmberTools 16 was used to prepare the topology and coordinates files. For that, the force field ff14SB [17] was specified, Na^+ and Cl^- ions were added to neutralize the system and achieve 0.10 M concentration of NaCl salt, and a truncated octahedral box of OPC3 waters [18] with the edges at least 10 Å away from the protein atoms was added.

The molecular dynamics (MD) simulations were carried out with PMEMD.CUDA [19,20] module of AMBER 16 [21]. In total, five minimization steps and twelve steps of equilibration dynamics were performed prior to the production MD. The first four minimization steps, composed of 2,500 cycles of the steepest descent algorithm followed by 7,500 cycles of conjugate gradient, were performed as follows: (*i*) in the first one, all the atoms of the protein and ligand were restrained with a 500 $kcal{\cdot}mol^{-1}{\cdot}Å^2$ harmonic force constant; (*ii*) in the following ones, only the backbone atoms of the protein and heavy atoms of the ligand were restrained, respectively, with 500, 125, and 25 $kcal{\cdot}mol^{-1}{\cdot}Å^2$ force constant. A fifth minimization step, composed of 5,000 cycles of the steepest descent and 15,000 cycles of conjugate gradient, was performed without any restraints. The subsequent MD simulations employed periodic boundary conditions, the particle mesh Ewald method for treatment of the long-range interactions beyond the 10 Å cutoff [22], the SHAKE algorithm [23] to constrain the bonds involving the hydrogen atoms, the Berendsen barostat[24] at

1 bar, the Langevin thermostat with collision frequency 1.0 ps^{-1}, and a time step of 2 fs. Equilibration dynamics were performed in twelve steps: (*i*) 20 ps of gradual heating from 0 to 310 K, under constant volume, restraining the protein atoms and ligand with 200 $kcal{\cdot}mol^{-1}{\cdot}Å^{2}$ harmonic force constant; (*ii*) ten MDs of 400 ps each, at constant pressure (1 bar) and constant temperature (310 K), with gradually decreasing the restraints on the backbone atoms of the protein and heavy atoms of the ligand with harmonic force constants of 150, 100, 75, 50, 25, 15, 10, 5, 1, and 0.5 $kcal{\cdot}mol^{-1}{\cdot}Å^{2}$; (*iii*) 400 ps of unrestrained MD at the same conditions as the previous restrained MDs. The energy and coordinates were saved every 10 ps. The production MDs were run for 500 ns using the same settings employed in the last equilibration step and performed in duplicate for each system, and three replicates were run. Each simulation was processed with *cpptraj* [25] module of AmberTools 16 to remove ions and solvent molecules, align the trajectory to the respective crystal structures by minimizing the root-mean-square deviation (RMSD) of the backbone atoms, and to calculate B-factors for each residue from the fluctuation of the respective backbone atoms. The trajectories were visualized using PyMol 2.3.2 [10]and VMD 1.9.1 [26].

EXPERIMENTAL SECTION

Protein expression and purification

All genes encoding DhaA variants used in this study were cloned in the pET21b plasmid in frame with C-terminal His-tag. The rationally designed mutations were introduced to the DhaA115 sequence using megaprimer PCR mutagenesis and their identity was verified by sequencing (Eurofins Genomics, Germany). Plasmids with genes encoding the FireProt and PROSS mutants were purchased from GeneArt (ThermoFisher Scientific, USA). Proteins were expressed from *E. coli* BL21 (DE3) and purified from cell lysates by immobilized metallo-affinity chromatography using HisTrap HP column (Cytiva, USA) charged with Ni^{2+} ions. The pure monomeric fraction was isolated by subsequent size exclusion chromatography using HiLoad 16/600 Superdex 75 pg column (Cytiva, USA). Protein purity was quantified using densitometric analysis of the SDS-PAGE gels and was found to be >99% for all variants if not stated otherwise in the text. All measurements were carried out in 50 mM potassium phosphate (PB) buffer pH 7.5 unless stated otherwise.

Hydrogen/deuterium exchange coupled with mass spectroscopy detection (HDX-MS)

Peptide mapping

Three hundred picomoles of DhA115 was mixed in 1:1 (v/v) ratio with 1 M glycine, pH 2.3 and injected on a pepsin column. Generated peptides were trapped and desalted by a Micro trap column (Luna Omega 5 um Polar C18 100 Å Micro Trap 20 x 0.3 mm) for 3 min at a 100 μL min^{-1} flow rate using isocratic pump delivering 0.4% formic acid in water. After 3 min digestion and desalting, peptides were separated on a C18 reversed phase column (Luna® Omega 1.6 μm Polar C18 100 Å ,100 x 1.0 mm) with a linear gradient 5-35% B in 26 min, where solvent A was 2% acetonitrile / 0.4% formic acid in water and solvent B 95% acetonitrile / 5% water / 0.4% formic acid. Protease

column, trap column and analytical column were placed in an icebox. TimsToF Pro Mass Spectrometer (Bruker Daltonics) operating in positive MS/MS mode was used for detection of peptides. Data was processed by DataAnalysis 5.3 software (Bruker Daltonics). MASCOT search engine was used for identification of peptides using a database containing sequence of DhaA115.

HDX

Hydrogen deuterium exchange was initiated by 10-fold dilution of the DhA115 in a deuterated buffer (50 mM PB, pD 7.5). The deuteration reaction was performed at 25°C, 50°C and 67°C. For comparison between 25°C and 67°C the incubation time points were 60 s, 180 s, 660 s and 1800 s for 67°C and 60 s, 660 s, 2074 s, 22813 s, 62216 s for 25°C (rate difference 34.565). Aliquots were quickly thawed and analyzed using the same system as described above. Peptides were separated by linear gradient 10-30% B in 18 min. 15T solariX FT-ICR mass spectrometer was operated in positive MS mode. Spectra of partially deuterated peptides were processed by Data Analysis 5.3 (Bruker Daltonics, Billerica, MA) and by in-house program DeutEx.

Temperature denaturation experiments

Differential scanning fluorimetry (DSF)

Changes in protein intrinsic fluorescence spectra excited at 266 nm were monitored during continuous heating of the samples from 20 to 95 °C at 0.5, 1, and 2°C/min scan rate using UNcle (Unchained labs, USA). The apparent melting temperature was evaluated from the midpoint of the average emission wavelength (i.e., Barycentric mean- BCM) curve. Each sample was analyzed in triplicate. The protein concentration was ca 0.17 mg/mL for all samples.

Circular dichroism (CD)

Changes in the secondary structure during temperature-induced unfolding were monitored using CD spectroscopy. Ellipticity at 227 nm was measured at 1 nm bandwidth with 0.25 s integration time while the temperature gradually increased at 1°C/min scan rate from 20 to 94 °C. The apparent melting temperature of each variant was assessed from the midpoint of the resulting sigmoidal curve. Each sample was measured in triplicate. The protein concentration was ca 0.17 mg/mL for all samples.

Differential scanning calorimetry (DSC)

Heat capacity changes of the proteins were recorded against dialysis buffer using VP-Capillary DSC (GE Healthcare, USA) from 25 to 90 °C at 1°C/min scan rate. Monomer samples at ca 0.8 mg/mL were exhaustively dialyzed against the 50 mM PB buffer pH 7.5. Buffer-subtracted data were normalized to protein concentration, followed by subtraction of a linear baseline connecting the pre- and post-transition signals. The apparent melting temperature of each variant was determined as the maximum of the heat capacity peak. Each sample was analyzed in triplicate.

Unfolding kinetics

The unfolding kinetics of the variants were measured by monitoring the ellipticity changes at 227 nm at different constant temperatures between 70 and 90 °C. The concentrated sample was diluted ~10 times to a pre-heated buffer to a final concentration of 0.17 mg/mL and immediately transferred to a pre-heated cuvette placed in the instrument measurement chamber. The whole process took approx. 5-10 s which accounts for the dead time of the measurement. The precise temperature of the solution was determined by a thermocouple inserted into the cuvette at the end of the measurement.

Global analysis

Thermal denaturation experiments of DhaA115, 222, 223, and 231 were analyzed globally using CalFitter 2.0 [27] webserver. The data measured using spectroscopy (CD, DSF), calorimetry (DSC), and unfolding kinetics (CD) were fitted globally to the two-step irreversible model of unfolding. The energy barriers of the first unfolding step $\Delta G^{\ddagger}$ (70 °C) and sum of the calorimetric enthalpies (ΔH_{cal}) of each step were used for comparison of the thermostabilities between the variants.

Chemical denaturation experiments

Thermodynamic stability of DhaA115, 222, 223, and 231 was determined from chemical denaturation experiments. Concentrated protein stocks were added to 48 aliquots of 50 mM phosphate buffer pH 7.5, 0.5 mM TCEP, pH 7.5, containing different concentrations of urea from 0 to 8.5 M to a final concentration of 0.1 mg.mL^{-1}. Samples were subsequently incubated at 25 °C until equilibrium between native and (partially-) unfolded states was reached (approx. 24 hours). Fluorescence emission spectra of the equilibrated samples between 300 and 400 nm were measured upon excitation at 280 nm using Synergy H4 Hybrid reader (Biotek, USA) in the 96 Well UV Transparent Plates (ThermoFisher, USA). Singular value decomposition (SVD) of the resulting spectral datasets was carried out using CalFitter 2.0 [28]. The first three most significant components of each variant were fitted globally in Origin 2021b (OriginLabs, USA) using least-square minimization algorithm to the three-state equilibrium model defined by the following scheme and equation 1:

$$N \overset{K_1}{\Leftrightarrow} I \overset{K_2}{\Leftrightarrow} D$$

(1) $y = y_N f_N + y_I f_I + y_D f_D$

where $y_{N,I,D}$ are the signals of native, intermediate, and denatured states, respectively, each described as linear function with individual parameters for slopes and intercepts. The $f_{N,I,D}$ denote the fractions of native, intermediate, and denatured states, respectively, and are calculated according to equations 2-4.

(2) $$f_N = 1/(1 + K_1 + K_2),$$

(3) $$f_I = K_1/(1 + K_1 + K_2)$$

(4) $$f_D = K_1 K_2/(1 + K_1 + K_2)$$

K_x are equilibrium constants parametrized according to equations 5-6.

(5) $$K_x = exp\left(-\left(m_x(C_{mx} - [urea])\right)/(RT)\right), x = 1, 2$$

(6) $$\Delta G_x = m_x C_{mx}, x = 1, 2$$

Here, ΔG_x is the Gibbs free energy of unfolding in the absence of urea, m_x, and C_{mx} are m-values and urea denaturation midpoints of each transition, respectively. The components were fitted globally to equation 1 which upon substitution of equations 2-6 yields ten parameters. The four parameters describing the transitions (m_1, m_2, Cm_1, Cm_2) were shared between the components, whilst the rest of them were allowed to vary for each component (in cases where possible, the slopes or intercepts defining the baselines were fixed to reduce the number of parameters).

Additionally, data were fitted to the two-state denaturation model according to the following scheme and equation 7.

$$N \overset{K}{\Leftrightarrow} D$$

(7) $$y = y_N f_N + y_D f_D ,$$

with fractions of states described by equations 8-9.

(8) $$f_N = 1/(1 + K),$$

(9) $f_D = 1 - f_N$

The relation of K constant and ΔG is given by equations 5 and 6. Data were fitted by equation 7 which upon substitutions of equations 8 and 9 and baseline parameters include six parameters.

Biochemical characterization

Specific activity measurements

The specific activity of selected variants towards 1,2-dibromoethane was determined using conventional colorimetric assay [29]. The dehalogenation reactions were carried out at 37 and 70 °C in 25-mL Reacti Flasks closed by Mininert Valves. Reaction mixture was composed of 12 mL of 100 mM glycine buffer (pH 8.6) and 12 μL of substrate (DBE). The reaction was initiated by the addition of the enzyme (~ 0.1 mg.mL^{-1} final concentration). The progress of the reaction was monitored over 30 minutes by withdrawing 1 mL aliquots from the reaction mixture in 4-minute intervals. At each time point, the reaction was terminated by addition of 0.1 mL of 35% nitric acid to the aliquot. The bromides formed during the reaction were detected spectrophotometrically upon reaction with mercuric thiocyanate and ferric ammonium sulphate using absorbance at 460 nm. The absorbance read-out was recalculated to the concentration of the product using calibration curve for bromides. The activity was calculated as the slope of the product concentration over time.

Temperature profiles and substrate specificity measurements

Both temperature and substrate specificity profiles were measured using the previously described capillary-based droplet microfluidic profile explorer (MicroPEX) [28]. enabling the characterization of specific enzyme activity in droplets for multiple enzyme variants in one run. The temperature profiles were measured towards 1,2-dibromoethane in 2.5-degree increments in the range of 55 °C to 70 °C. The substrate specificity of individual enzyme variants was measured

towards 27 representative halogenated substrates (**Supplementary Table 9**), previously chosen to validate the microfluidic device [30], at 60 °C which is close to the temperature of maximal activity (T_{max}) of all the variants and still within the technical limits of the pH assay.

MicroPEX measurements were performed as follows. The reaction solutions consisted of selected enzymes, weak buffer (1 mM HEPES, 20 mM Na_2SO_4, pH 8.2) and a complementary fluorescent indicator 8-hydroxypyrene-1,3,6-trisulfonic acid (50 μM HPTS). Droplets were generated using Mitos Dropix (Dolomite, UK). A custom sequence of droplets (150 nL aqueous phase, 300 nL oil spacing) was generated using negative pressure (microfluidic pump). Generated droplets were subsequently moved through a polyethene tubing to the incubation chamber, where the halogenated substrate was delivered to the droplets via a combination of microdialysis and partitioning between the oil (FC40) and the aqueous phase. The fluorescence signal from the droplets was measured using an optical setup with an excitation laser (450 nm), a dichroic mirror with a cut-off at 490 nm filtering the excitation light, and a Si-detector. Small changes in pH during dehalogenation were detected using the fluorescence pH assay. Reaction progress was analyzed as an end-point measurement recorded after the passing of 10 droplets/sample through the incubation chamber. The reaction time was approx. 4 min. The raw fluorescence signal of every single measurement was at first processed by a LabView-based code (National Instruments, USA) developed in-house. The peaks were assigned to the particular sample using this software, and the mean signal was calculated. Then, the output XLS file containing the mean signal values of every sample type (calibration, enzyme activity, buffer, blank buffer, and blank enzyme) for a particular dataset is served as an input for the MATLAB script (MathWorks, USA) that calculated the specific enzymatic activities using the principle described previously [31]. The activities were classified as “not determined” whenever the measured product concentration was below the limit

of detection (LOD – 3 times the standard deviation of the noise signal). Each substrate had a different calibration curve, so the LOD product concentration was in the range of 10-100 µM). For the substrates 1,2-dichloroethane (S37), 1,2-dichloropropane (S67), 1,2-dibromopropane (S72), (Bromomethyl)cyclohexane (S119), 1,2-dibromo-3-chloropropane (S155), and 3-chloro-2-methylpropene (S209) it was not possible to obtain even calibration data, so these substrates are unsuitable for measurement at 60 °C.

Steady-state kinetics with 1,2-dibromoethane

Steady-state kinetics of DhaA222, 223, and 231 towards 1,2-dibromethane were measured by the multiple-titration calorimetric assay using VP-ITC isothermal titration calorimeter (MicroCal, USA) at 25, 37, 50, and 60 °C. All reactions were carried out in the 100 mM glycine buffer pH 8.6. The substrate solution was prepared by dissolving 15 µL of DBE in 4 mL of the reaction buffer for 30 minutes at 37 °C. Before each titration, 100 µL of the substrate mixture was extracted by 900 µL of acetone containing internal standard (1,2,3-trichloropropane) and analyzed by gas chromatography to quantify the amount of dissolved substrate. Proteins were thoroughly dialyzed and diluted to the reaction buffer to final concentrations ranging between $3.125.10^{-4}$ and 0.1 mg/ml. The substrate solution in the injector was titrated to the sample cell containing protein solution in twenty-eight 2.5 µL injections with 150 s delay between them. The rates of heat change at every injection were recalculated to the reaction velocity using the value of heat released during the total conversion of DBE. This value was determined in the separate experiment in which 10 µL of substrate was titrated into the reaction cell filled with 1.4 mL of enzyme in 8 injections with 3000 s spacing between them. The resulting peaks were integrated, and the integral values averaged to yield the total heat released during complete conversion of DBE. The resulting steady-

state kinetics data were fitted by a nonlinear regression to the model involving substrate inhibition (equation 10) using Origin 2021b (OriginLab, USA).

(10) $$v = \frac{V_{max}[S]}{K_M + [S](1 + \frac{[S]}{K_{si}})}$$

Where v and v_{max} denote the observed and maximal rates of the reaction, respectively, [S] is the concentration of substrate, K_m is the Michaelis-Menten constant, and K_{si} is the substrate inhibition constant.

Thermodynamic analysis

The thermodynamic parameters of the catalytic rate (k_{cat}) and equilibrium (K_m, K_{si}) constants were obtained from the Eyring and van't Hoff plots, respectively, using equations 11 and 12:

(11) $$ln(k_{cat}/T) = -\Delta H^{\ddagger}/RT + ln(k_B/h) + \Delta S^{\ddagger}/R$$

(12) $$ln([S]_{ref}/K_{eq}) = -\Delta H/RT + \Delta S/R$$

, where T is the thermodynamic temperature in Kelvins, R is the universal gas constant, k_B is the Boltzmann constant, h – Planck constant, $\Delta H^{\ddagger}$ and $\Delta S^{\ddagger}$ are the activation enthalpy and entropy, respectively, $[S]_{ref}$ is the reference substrate concentration (set to 1 mM in the calculations), K_{eq} is either K_m or K_{si}, ΔH and ΔS are the standard enthalpy and entropy, respectively. The enthalpy was assumed to be temperature independent ($\Delta C_p = 0$). The Gibbs free energy (ΔG) and was then calculated according to the equation 13.

(13) $$\Delta G = \Delta H - T\Delta S$$

Structural analysis of DhaA222 and DhaA223

Crystallization experiments

Diffraction-quality crystals of the DhaA223 enzyme were obtained at 20°C by mixing equal volumes of DhaA223 protein (10 mg mL^{-1}) with reservoir solution composed of 20% PEG 3350,

0.2 M ammonium sulphate and 0.1 M Bis-tris buffer (pH 5.5) and crystallized using the sitting-drop vapor diffusion technique. Crystals of the DhaA231 enzyme were obtained at 20°C by mixing equal volumes of DhaA231 protein (10 mg mL^{-1}) with reservoir solution composed of 10% PEG 8000, 0.2 M magnesium chloride and 0.1 M Tris buffer (pH 7) and crystallized using the sitting-drop vapor diffusion technique. After 4 to 6 days, the crystals so grown were briefly transferred into corresponding reservoir solution supplemented with 20% glycerol and slash-frozen in liquid nitrogen.

Structure determination and refinement

The crystallographic data were collected at Swiss Light Source synchrotron (beamline PXI) and processed using XDS [32] and Aimless [33]. Initial phases were solved by molecular replacement using Phaser [34] implemented in the Phenix software package [35]. The structure of DhaA115 (PDB ID: 6SP5) was employed as a search model for replacement. The structural refinements were carried out with multiple cycles in the phenix.refine [35] and manual model building was performed in Coot [36]. Crystals structures of DhaA223 and DhaA231 were solved to resolutions of 1.5 Å and 1.3 Å in a $P12_11$ and $P2_12_12_1$ space group, respectively. The final models were validated using tools provided by Coot [36] and Phenix [35]. Visualization of structural data was done with PyMOL [10]. Atomic coordinates and structure factors of the DhaA223 and DhaA231 enzymes were deposited in the Protein Data Bank under the PDB ID codes 8OE2 and 8OE6.

References

1. Craig, D. B.; Dombkowski, A. A.; Disulfide by Design 2.0: a web-based tool for disulfide engineering in proteins. *BMC Bioinformatics* **2013**; 14 (346), DOI: 10.1186/1471-2105-14-346

2. Markova, K.; Chmelova, K.; Marques, S. M.; Carpentier, P.; Bednar, D.; Damborsky, J.; Marek, M.; Decoding the intricate network of molecular interactions of a hyperstable engineered biocatalyst. *Chem Sci.* **2020**, 11 (41), 11162–11178. DOI: 10.1039/d0sc03367g

3. Kellogg, E. H.; Leaver-Fay, A.; Baker, D. Role of conformational sampling in computing mutation-induced changes in protein structure and stability: Conformational Sampling in Computing Mutation-Induced Changes. *Proteins* **2011**, 79 (3), 830–838. DOI: 10.1002/prot.22921

4. Song, Y.; Tyka, M.; Leaver-Fay, A.; Thompson, J.; Baker, D. Structure-guided forcefield optimization. *Proteins* **2011**, 79, 1898–1909.

5. O'Meara, M. J.; Leaver-Fay, A.; Tyka, M. D.; Stein, A.; Houlihan, K.; DiMaio, F.; Bradley, P.; Kortemme, T.; Baker, D.; Snoeyink, J.; Kuhlman, B. Combined Covalent-Electrostatic Model of Hydrogen Bonding Improves Structure Prediction with Rosetta. *J Chem Theory Comput*. **2015** 11 (2), 609–622. DOI: 10.1021/ct500864r.

6. Sumbalova, L.; Stourac, J.; Martinek, T.; Bednar, D.; Damborsky, J. HotSpot Wizard 3.0: web server for automated design of mutations and smart libraries based on sequence input information. *Nucleic Acids Res*. **2018**, 46 (W1), W356–W362. DOI: 10.1093/nar/gky417

7. Sumbalova, L.; Stourac, J.; Martinek, T.; Bednar, D.; Damborsky J.; HotSpot Wizard 3.0: web server for automated design of mutations and smart libraries based on sequence input information. *Nucleic Acids Res.* **2018**, 46, W356–W362.

8. Musil, M.; Stourac, J.; Bendl, J.; Brezovsky, J.; Prokop, Z.; Zendulka, J.; Martinek, T.; Bednar, D.; Damborsky, J. FireProt: web server for automated design of thermostable proteins. *Nucleic Acids Res.* **2017**, 45 (W1),W393–399. DOI: 10.1093/nar/gkx285.

9. Goldenzweig, A.; Goldsmith, M.; Hill, S. E.; Gertman, O.; Laurino, P.; Ashani, Y.; Dym, O.; Unger, T.; Albeck, S.; Prilusky, J.; Lieberman, R. L.; Aharoni, A.; Silman, I.; Sussman, J. L.; Tawfik, D. S.; Fleishman, S. J. Automated Structure- and Sequence-Based Design of Proteins for High Bacterial Expression and Stability. Mol Cell. 2016, 63 (2), 337–346. DOI: 10.1016/j.molcel.2016.06.012

10. The PyMOL Molecular Graphics System, Version 2.3.2, Schrödinger, LLC, 2019

11. Ittisoponpisan, S.; Islam, S. A.; Khanna, T.; Alhuzimi, E.; David, A.; Sternberg, M. J. E. Can Predicted Protein 3D Structures Provide Reliable Insights into whether Missense Variants Are Disease Associated? *J Mol Biol.* **2019**, 431 (11), 2197–2212. DOI: 10.1016/j.jmb.2019.04.009

12. Fernandez-Escamilla, A.M.; Rousseau, F.; Schymkowitz, J.; Serrano, L.; Prediction of sequence-dependent and mutational effects on the aggregation of peptides and proteins. *Nat. Biotechnol.* **2004**, 22, 1302–1306. DOI: 10.1038/nbt1012

13. Kuriata, A.; Iglesias, V.; Pujols, J.; Kurcinski, M.; Kmiecik, S.; Ventura, S.; Aggrescan3D (A3D) 2.0: prediction and engineering of protein solubility. *Nucleic Acids Res.* **2019** 47, W300–W307. DOI: 10.1093/nar/gkz321.

14. Shroff, R.; Cole, A. W.; Diaz, D. J.; Morrow, B. R.; Donnell, I. Annapareddy, A.; Gollihar, J.; Ellington, A. D.; Thyer, R. Discovery of Novel Gain-of-Function Mutations Guided by Structure-Based Deep Learning. *ACS Synth Biol.* **2020**, 9 (11), 2927–2935. DOI: 10.1021/acssynbio.0c00345

15. Case, D. A.; Cheatham, T. E., 3rd; Darden, T.; Gohlke, H.; Luo, R.; Merz, K. M., Jr.; Onufriev, A.; Simmerling, C.; Wang, B.; Woods, R. J., The Amber biomolecular simulation programs. *J Comput Chem* **2005**, 26, 1668-1688. DOI: 10.1002/jcc.20290

16. Gordon, J. C.; Myers, J. B.; Folta, T.; Shoja, V.; Heath, L. S.; Onufriev, A., H++: a server for estimating pKas and adding missing hydrogens to macromolecules. *Nucleic Acids Res* **2005**, 33 (Web Server), W368-W371. DOI: 10.1093/nar/gki464

17. Maier, J. A.; Martinez, C.; Kasavajhala, K.; Wickstrom, L.; Hauser, K. E.; Simmerling, C., ff14SB: Improving the Accuracy of Protein Side Chain and Backbone Parameters from ff99SB. *J Chem Theory Comput* **2015**, 11, 3696-3713. DOI: 10.1021/acs.jctc.5b00255

18. Izadi, S.; Onufriev, A. V., Accuracy limit of rigid 3-point water models. *J. Chem. Phys.* **2016**, 145, 074501. DOI: 10.1063/1.4960175

19. Götz, A. W.; Williamson, M. J.; Xu, D.; Poole, D.; Le Grand, S.; Walker, R. C., Routine Microsecond Molecular Dynamics Simulations with AMBER on GPUs. 1. Generalized Born. *J Chem Theory Comput* **2012**, 8, 1542-1555. DOI: 10.1021/ct200909j

20. Le Grand, S.; Götz, A. W.; Walker, R. C., SPFP: Speed without compromise—A mixed precision model for GPU accelerated molecular dynamics simulations. *Comput Phys Commun* **2013**, 184, 374-380. DOI: 10.1016/j.cpc.2012.09.022

21. Case, D. A.; Berryman, J.; Betz, R.; Cerutti, D.; Cheatham III, T.; Darden, T.; Duke, R.; Giese, T.; Gohlke, H.; Goetz, A.; Homeyer, N.; Izadi, S.; Janowski, P.; Kaus, J.; Kovalenko, A.; Lee, T. S.; LeGrand, S.; Li, P.; Luchko, T.; Luo, R.; Madej, B.; Mertz, K. M.; Monard, G.; Needham, P.; Nguyen, H.; Nguyen, H. T.; Omelyan, I.; Onufriev, A.; Roe, D.; Roitberg, A.; Salomon-Ferrer, R.; Simmerling, C. L.; Smith, W.; Swails, J.; Walker, R. C.; Wang, J.; Wolf, R. M.; Wu, X.; York, D. M.; Kollman, P. A., AMBER 2015. University of California: San Francisco, **2015**.

22. Darden, T.; York, D.; Pedersen, L., Particle mesh Ewald: An N· log (N) method for Ewald sums in large systems. *J Chem Phys* **1993**, 98, 10089-10092. DOI: 10.1063/1.464397

23. Ryckaert, J.-P.; Ciccotti, G.; Berendsen, H. J., Numerical integration of the cartesian equations of motion of a system with constraints: molecular dynamics of n-alkanes. *J Comput Phys* **1977**, 23, 327-341. DOI: 10.1016/0021-9991(77)90098-5

24. Berendsen, H. J. C.; Postma, J. P. M.; van Gunsteren, W. F.; DiNola, A.; Haak, J. R., Molecular dynamics with coupling to an external bath. *J Chem Phys* **1984**, 81, 3684–3690. DOI: 10.1063/1.448118

25. Roe, D. R.; Cheatham, T. E., PTRAJ and CPPTRAJ: Software for Processing and Analysis of Molecular Dynamics Trajectory Data. *J Chem Theory Comput* **2013,** *9*, 3084-3095. DOI: 10.1021/ct400341p

26. Humphrey, W.; Dalke, A.; Schulten, K., VMD: visual molecular dynamics. **1996,** *14*, 33-38.

27. Mazurenko, S.; Stourac, J.; Kunka, A.; Nedeljkovic, S.; Bednar, D.; Prokop, Z.; Damborsky, J.; CalFitter: A Web Server for Analysis of Protein Thermal Denaturation Data. *Nucleic Acids Res.* **2018**, 46 (W1), W344–349. DOI: 10.1093/nar/gky358

28. Kunka, A.; Lacko, D.; Stourac, J.; Damborsky, J.; Prokop, Z.; Mazurenko, S. CalFitter 2.0: Leveraging the power of singular value decomposition to analyze protein thermostability. *Nucleic Acids Res.* **2022**, 50 (W1), W145-W151. DOI: 10.1093/nar/gkac378

29. Iwasaki, I., Satori, U., Takejiro, O., New colorimetric determination of chloride using mercuric thiocyanate and ferric ion. *BCSJ* **1952**, 25 (3), 226, https://doi.org/10.1246/bcsj.25.226

30. Buryska, T.; Vasina, M.; Gielen, F.; Vanacek, P.; van Vliet; Jezek, L.; Pilat, Z.; Zemanek, P.; Damborsky, J.; Hollfelder, F.; Prokop, Z. Controlled oil/water partitioning of hydrophobic substrates extending the bioanalytical applications of droplet-based microfluidics. *Anal. Chem.* **2019**, 91, 15, 10008–10015. DOI: 10.1021/acs.analchem.9b01839.

31. Vasina, M.; Vanacek, P.; Damborsky, J.; Prokop, Z.; Exploration of enzyme diversity: High-throughput techniques for protein production and microscale biochemical characterization. In *Methods in Enzymology*. Elsevier, **2020**; p. 51–85. DOI: 10.1016/bs.mie.2020.05.004.

32. Kabsch, W., XDS. *Acta Crystallographica D* **2010**, 66, 125-132. DOI: 10.1107/S0907444909047337.

33. Evans, P.R.; Murshudov, G.N.; How good are my data and what is the resolution? *Acta Crystallographica D* **2013**, 69, 1204-1214. DOI: 10.1107/S0907444913000061

34. McCoy, A. J.; Grosse-Kunstleve, R. W.; Adams, P. D.; Winn, M. D.; Storoni, L. C.; Read, R. J., J. Phaser crystallographic software. *Appl. Cryst.* **2007**, 40, 658-674. DOI: 1 0.1107/S0021889807021206

35. Adams, P.D.; Afonine, P.V.; Bunkóczi, G.; Chen, V. B.; Davis I.W.; Echols, N., Headd, J. J.; Hung, L.; Kapral, G. J.; Grosse-Kunstleve, R. W.; McCoy, A. J.; Moriarty, N. W.; Oeffner, R.; Read, R. J.; Richardson, D. C.; Richardson, J. S.; Terwilliger, T. C.; Zwart, P. H. PHENIX: a comprehensive Python-based system for macromolecular structure solution. *Acta Crystallogr D* **2010**, 66, 213–221. DOI: 10.1107/S0907444909052925

36. Emsley P.; Cowtan K. Coot: model-building tools for molecular graphics. *Acta Crystallogr D* **2004**. D60, 2126-2132 DOI: 10.1107/S0907444904019158
